# Epigenetic Changes Regulating Epithelial–Mesenchymal Plasticity in Human Trophoblast Differentiation

**DOI:** 10.3390/cells14130970

**Published:** 2025-06-24

**Authors:** William E. Ackerman IV, Mauricio M. Rigo, Sonia C. DaSilva-Arnold, Catherine Do, Mariam Tariq, Martha Salas, Angelica Castano, Stacy Zamudio, Benjamin Tycko, Nicholas P. Illsley

**Affiliations:** 1 Department of Obstetrics and Gynecology, Institute for Health Data Science Research, AI.Health4All Center, University of Illinois Chicago, Chicago, IL 61607, USA; weackerm@uic.edu; 2 Hackensack Meridian Health Center for Discovery and Innovation, Nutley, NJ 07110, USA; mauriciorigo@tyckolab.com (M.M.R.); catherine.do2@nyulangone.org (C.D.); mariam.tariq@hmh-cdi.org (M.T.); martha.salas@hmh-cdi.org (M.S.); angelica.castano@hmh-cdi.org (A.C.); benjamintycko@gmail.com (B.T.); 3 Department of Obstetrics and Gynecology, Hackensack University Medical Center, Hackensack, NJ 07601, USA; soninhausa@gmail.com (S.C.D.-A.); placresgp@gmail.com (S.Z.)

**Keywords:** trophoblast, differentiation, epithelial–mesenchymal transition, DNA methylation

## Abstract

The phenotype of human placental extravillous trophoblast (EVT) at the end of pregnancy reflects both differentiation from villous cytotrophoblast (CTB) and later gestational changes, including loss of proliferative and invasive capacity. Invasion abnormalities are central to major obstetric pathologies, including placenta accreta spectrum, early onset preeclampsia, and fetal growth restriction. Characterization of the normal differentiation processes is, thus, essential for the analysis of these pathologies. Our gene expression analysis, employing purified human CTB and EVT cells, demonstrates a mechanism similar to the epithelial–mesenchymal transition (EMT), which underlies CTB–EVT differentiation. In parallel, DNA methylation profiling shows that CTB cells, already hypomethylated relative to non-trophoblast cell lineages, show further genome-wide hypomethylation in the transition to EVT. A small subgroup of genes undergoes gains of methylation (GOM), associated with differential gene expression (DE). Prominent in this GOM-DE group are genes involved in epithelial–mesenchymal plasticity (EMP). An exemplar is the transcription factor *RUNX1*, for which we demonstrate a functional role in regulating the migratory and invasive capacities of trophoblast cells. This analysis highlights epigenetically regulated genes acting to underpin the epithelial–mesenchymal plasticity characteristic of human trophoblast differentiation. Identification of these elements provides important information for the obstetric disorders in which these processes are dysregulated.

## 1. Introduction

Differentiation of cytotrophoblast (CTB) into invasive extravillous trophoblast (EVT) is a crucial element in human placentation. The EVT at the distal ends of chorionic villi attach the placenta to the decidua, acting as anchor points for the placenta [1]. EVT cells moving into the uterus from the implantation site early in pregnancy form plugs in the spiral arteries, preserving a low oxygen environment around the conceptus and promoting trophoblast proliferation in the rapidly growing placenta [2]. EVT then acts as a key cell type in remodeling the maternal spiral arteries to efficiently deliver maternal blood to the intervillous space [1].

Elucidation of the differentiation and invasion processes, whereby anchored CTB is transformed into invasive EVT, is central to our understanding of placental development and major obstetric pathologies. One such condition is early-onset preeclampsia (PE), in which EVT invasion is more limited than in normal pregnancy, leading to decreased spiral artery remodeling, reduced uteroplacental blood flow, increased oxidative stress, maternal endothelial damage, and clinical sequelae [3,4]. At the other extreme is the placenta accreta spectrum (PAS), where EVT invades more deeply into the uterus, with loss of decidual tissue and direct adherence of villous tissue to the myometrium. This also results in atypical remodeling of deeper uterine vessels, possibly as deep as the radial arteries [5], leading to potentially fatal hemorrhage at delivery when PAS goes undetected. In both disorders, invasion is abnormal, raising questions regarding the invasion mechanism and its control.

Prior research has suggested that a key molecular mechanism governing trophoblast differentiation and invasion is an epithelial–mesenchymal transition mechanism (EMT [6,7,8]). EMT is common to multiple processes, such as gastrulation, wound healing, and cancer metastasis, whereby anchored epithelial cells are converted to an invasive, mesenchymal phenotype [9]. We have demonstrated changes in gene expression indicative of an EMT-like mechanism in both first- and third-trimester EVT [10,11]. While first-trimester EVT occupies a relatively mesenchymal position on the EMT spectrum, term EVT appears to show a degree of regression towards the epithelial pole [12]. This epithelial–mesenchymal plasticity (EMP) reflects the loss of EVT invasiveness observed in mid- and late pregnancy. The reduction in invasion later in gestation is part of normal establishment of the placenta, a process which may go awry in pathologies such as preeclampsia, growth restriction, and accreta. These events prompted us to investigate the differentiation process in depth, via measurements of gene expression and DNA methylation.

## 2. Materials and Methods

### 2.1. Placental Tissue and Cell Isolation

Tissue used in this report was obtained from normal, term (≥39 weeks’ gestational age) pregnancies, delivered by elective caesarean without labor. Tissue was transported to the laboratory, on ice, and processed within 20 min of delivery. Excluded were pregnancies with medical or obstetric complications, mothers under 18 years of age, or with a BMI ≥ 30.

EVT and CTB were isolated from these placentae for RNA sequencing and DNA methylation analysis as previously described [11]. A thin (3–4 mm) layer of tissue from the maternal-facing basal plate of the placenta was removed immediately following delivery. Tissue (~20 g) was washed ×2 in phosphate-buffered saline (PBS), then further dissected into small pieces (2–4 mm) and washed ×2 in PBS. Following dissection, the placental tissue was incubated at 37 °C in calcium- and magnesium-free Hank’s Balanced Salt Solution (HBSS) containing 10 mM Hepes, pH 7.4, with 0.05% trypsin (ThermoFisher Scientific, Waltham, MA, USA), 0.1% dispase (Worthington Biochemical, Lakewood, NJ, USA), and 12 U/mL Universal Nuclease (ThermoFisher). Tissue was incubated for 20 min in the digestion mix, and then the supernatant was decanted through a 100-μm filter into FBS (final concentration 10%; Atlanta Biologicals, Flowery Branch, GA, USA) and placed on ice. Another aliquot of the digestion solution was added to the remaining tissue, and the incubation and filtration steps were repeated, ×2. Cells were pelleted by centrifugation (10 min, 1200× *g*, 4 °C) and resuspended in Separation Buffer (SB; calcium- and magnesium-free HBSS containing 2 mM EDTA, 0.5% BSA, pH 7.4 at 4 °C). Lysis of red blood cells in the cell suspension was performed by rapid mixing of the cell suspension with an NH_4_Cl solution to obtain a final solution containing 155 mM NH_4_Cl, 10 mM KHCO_3_, 0.1 mM EDTA, pH 7.4. Following incubation of this lysis mixture for 10 min at room temperature, the cells were passed serially through 70 and 40 μm filters before centrifugation at 1200× *g* for 10 min (4 °C; unless otherwise stated, all subsequent steps were carried out at 4 °C). Cells were resuspended in Dulbecco’s Modified Eagle Medium (DMEM) without calcium, containing 10% FBS, 1% penicillin/streptomycin, and adjusted to pH 7.4 (DMEM buffer). After a further wash in DMEM buffer, the cell mixture was divided into two aliquots, which were incubated with either a mouse monoclonal anti-HLA-G antibody coupled to R-phycoerythrin (HLA-G-PE, 1:500, clone MEM G/9; Abcam, Waltham, MA, USA) for isolation of EVT, or with a mouse monoclonal anti-integrin ß4 antibody coupled to R-phycoerythrin (ITGß4-PE, 1:500, clone 58XB4, BioLegend, San Diego, CA, USA) for isolation of CTB. The cell mixture was incubated with the antibody for 30 min. Labeled cells were washed ×2 in DMEM buffer (400× *g*, 5 min), resuspended, counted, and the volume was adjusted to give 10^7^ cells/100 μL. Cells were incubated with anti-PE microbeads (10 μL microbeads/10^7^ cells; Miltenyi, San Diego, CA, USA) for 20 min, washed, and resuspended in DMEM buffer. Isolation of HLA-G- and ITGß4-labeled cells was performed on an AutoMACS immunomagnetic cell separator (Miltenyi) using a double-column, positive selection procedure, as specified by the manufacturer. In addition to the CTB isolated from the basal plate, CTB was also prepared from villous tissue, obtained from the area midway between the placental basal and chorionic plates. Cells were prepared in the same way as the CTB isolated from the basal plate and are referred to as villous CTB (vCTB).

After separation, cells in the positive-selection fractions were counted, spun down (400× *g*, 5 min), and cell samples were taken to assess purity using flow cytometry. These samples were incubated with Sytox Green (30 nM, 20 min, room temperature; ThermoFisher) for assessment of cell death. Labeled cells were analyzed by flow cytometry using a Cytomics FC500 flow cytometer (Beckman-Coulter, Indianapolis, IN, USA). After analysis by flow cytometry, cells labeled by the anti-HLA-G antibody were found to comprise 95.6 ± 1.2% of the EVT fraction (*n* = 9). Cells labeled by the anti-integrin ß4 antibody comprised 96.8 ± 0.6% of the CTB fraction (*n* = 9).

Cells were aliquoted for preparation of RNA (0.5 − 1.0 × 10^6^ cells/sample) or DNA (1.0 × 10^6^ cells/sample) and washed in PBS. Cells for preparation of RNA were pelleted and then lysed immediately in Qiazol (Qiagen, Valencia, CA, USA) and frozen at −80 °C. Cells for DNA preparation were directly frozen at −80 °C after pelleting.

For the fresh/incubated comparison, freshly isolated EVT were divided into two aliquots. The first aliquot (1 × 10^6^ cells) was immediately extracted in Qiazol, as described above. The second aliquot was plated onto fibronectin-coated 6-well plates (1 × 10^5^/well) immediately after isolation and cultured for 72 h in DMEM/F12 containing 1% penicillin/streptomycin, 10% FBS, and 50 µg/mL gentamicin (ThermoFisher). Medium was replaced after 24 and 48 h, and cells were extracted into Qiazol after 72 h.

### 2.2. siRNA Treatment

JEG3 choriocarcinoma cells (ATCC, Manassas, VA, USA) were cultured at 37 °C in DMEM/F12 containing 1% penicillin/streptomycin, 10% FBS (DMEM/F12). Cells (3 × 10^5^) were plated in each well of a 6-well plate and cultured to obtain a confluence of ~70%. Cells were transfected with 30 nM siRNA (Silencer Select, ThermoFisher) directed against RUNX1 (Silencer^®^ siRUNX1; #AM16708, assay ID 106564), or a negative control (siNEG; negative control #1), made up in Advanced DMEM/F12 (LDP, Cat# 12634010), and mixed with RNAiMAX (ThermoFisher) diluted in Advanced DMEM/F12 according to the manufacturer’s protocol. Following the addition of the siRNA/RNAiMAX mixture, cells were incubated overnight in Advanced DMEM/F12 without antibiotics and then switched to complete DMEM/F12 for a total incubation period of 48 h. At the end of the incubation period, cells were used in the migration/scratch assay and were also extracted, after washing, into Qiazol for qRT-PCR or into RIPA lysis buffer for Western blotting.

### 2.3. Cell Migration and Invasion Assays

The migration of JEG3 cells was measured using a scratch assay. After a 48-h transfection, JEG3 cells (siRUNX1 or siNEG) were plated in a 12-well plate and grown to 70–80% confluence (18–24 h). Once at the requisite confluence, the cell layer was scraped in a straight line using a 1 mm sterile pipette tip. After scratching, the cell monolayer was washed to remove detached cells, then replenished with fresh medium. Wells were imaged by phase-contrast microscopy at 0, 24, 48, and 72 h.

For the Transwell invasion assays, Matrigel was thawed on ice and prepared at a concentration of 0.2 mg/mL, diluted in Advanced-DMEM/F12. Cold Fluoroblok inserts (VWR, Cat# 62406-504) were coated with 100 µL of Matrigel and incubated at 37 °C overnight to allow the Matrigel to gel. Cell suspensions (~60,000 cells per well; siNEG or siRUNX1 cells) were added to each of the four wells. Subsequently, 0.5 mL of DMEM/F12 was added to the lower reservoir of each well, and the plate was incubated for 48 h. After incubation, calcein AM stock (Life Technologies, Santa Clara, USA, Cat# C1429; 1 mM in DMSO) was diluted to 2µM in warm HBSS/Hepes. The medium from the bottom reservoir was removed by aspiration, and the reservoir was washed with HBSS/Hepes. Subsequently, 0.5 mL of the diluted calcein AM solution was added to the bottom reservoir of the wells, and the plate was incubated at 37 °C for 60 min. Total fluorescence was read from the bottom of the plates using a BioTek Synergy HT plate reader with excitation of ~488 nm and an emission of ~520 nm.

### 2.4. RNA Sequencing

RNA was extracted using the miRNeasy Micro kit (Qiagen) according to the manufacturer’s instructions. Genomic DNA was enzymatically digested by DNase I treatment, and total RNA was captured by column purification. Both RNA concentration and integrity were quantified on an RNA 6000 Nano Chip using the Agilent 2100 Bioanalyzer (Agilent Technologies, Santa Clara, CA, USA). These cell fractions yielded RNA with RIN scores of 8.7 ± 0.3 (EVT) and 9.4 ± 0.2 (CTB), sufficient quality for analysis.

RNA sequencing was performed by the Genomics Center at Rutgers—New Jersey Medical School. Prior to RNA sequencing, the quality of the RNA was checked using Agilent Tapestation. The RNA was ribosome-depleted using the Ribo-Zero kit (Illumina, San Diego, CA, USA). Ribosome-depleted RNA was used to make cDNA libraries using the NEBNext Ultra II RNA Library preparation kit for Illumina and NEBNext Multiplex Oligos for Illumina as per the manufacturer’s protocol (New England Biolabs, Ipswich, MA, USA). Briefly, ribosome-depleted RNA was chemically fragmented and reverse-transcribed to make cDNA. The resulting cDNA was end-repaired and A-tailed. Adapters were ligated, and libraries were PCR amplified using indexed primers. Libraries were then purified using Ampure XP beads (Beckman Coulter) and quantified using TapeStation and Qubit. Sequencing was performed on an Illumina NextSeq 500 with a 1 × 75 configuration. The resulting raw data (bcl files) were converted to Fastq files and demultiplexed using Illumina software (bcl2fastq).

### 2.5. RNA Sequencing Data Analysis

Quality control on raw Fastq files was performed using FastQC v0.11.8 (www.bioinformatics.babraham.ac.uk, accessed on 5 January 2019), and reads were mapped to the hg38 genome build with STAR v2.7.4a [13] using the following parameters: --runMode alignReads; --outSAMtype BAM Unsorted; --readFilesCommand zcat; --genomeLoad LoadAndKeep; --clip3pAdapterSeq AGATCGGAAGAGC; --clip3pAdapterMMp 1; –-outFilterScoreMinOverLread 0.3; –-outFilterMatchNminOverLread 0.3. Feature counts were generated using SAMtools v1.10 [14] in combination with HTSeq v0.11.2 [15]; specifically, the output of the “samtools view” command was piped to the “htseq-count” command with the –stranded = reverse parameter in the Python v3.6.1 environment. Human Genome Organization Gene Nomenclature Committee (HGNC) symbols were updated using the R package HGNChelper v0.8.1 [16].

### 2.6. Differential Abundance Analysis

Statistical testing for differential transcript abundance was performed with DESeq2 v1.34.0 [17] in R using models that included the cell type and subject identifier (placenta of origin). Assigned fetal sex was considered separately since its inclusion, together with subject information, provided a design formula that was not full rank, such that models could not be fit. Statistical significance was considered to be a false discovery rate (FDR) < 0.05 using the Benjamini–Hochberg procedure.

### 2.7. Functional Enrichment Analysis and Visualization

Functional enrichment was assessed by overrepresentation analysis (hypergeometric test) using the enricher() function in the clusterProfiler v4.2.2 R package [18]. Specifically, we tested for enrichment of the Hallmark gene sets (v7.4) obtained from the Molecular Signatures Database (MSigDB) collections using differentially abundant transcripts (FDR < 0.05) compared to the background of all mapped transcripts; parameters included gene sets between 10 and 250 members in size, a threshold *p*-value of 0.05, and the Benjamini–Hochberg procedure for *p*-value adjustment.

The GeneTonic v1.6.1 workflow [19] was used for interactive visualization of the enrichment results. In this environment, the expression and statistical results tables generated by DESeq2 were combined with the enrichment analysis results obtained from clusterProfiler and an annotation data table acquired using the AnnotationDbi v1.56.2 package and the Bioconductor annotation package org.Hs.eg.db v3.14.0. Gene-gene set bipartite network graphs were produced using the ggs_graph() function and visualized using the visIgraph() function in the visNetwork library (https://CRAN.R-project.org/package=visNetwork, accessed on 5 January 2019). Individual heatmaps for enriched hallmark pathways were generated using the gs_heatmap() function. Radar plots and volcano plots were created with the gs_radar() and gs_volcano() functions, respectively. Alternate renderings of these results were generated with Prism v.10.4.1 (GraphPad Software, San Diego, CA, USA). Biological process enrichment analysis was also performed using GORILLA (Gene Ontology enRIchment anaLysis and visuaLizAtion tool [20,21]). A list of target genes was utilized in conjunction with a background list comprising all mapped transcripts.

### 2.8. DNA Methylation

Analysis of DNA methylation was performed as described previously [22], using Illumina Infinium Methylation EPIC v1 BeadChips. High molecular weight DNA was prepared from the frozen cell pellets by standard SDS/proteinase-K lysis followed by precipitation in 80% isopropanol with glycogen as a carrier. Genomic DNA quality was evaluated by gel electrophoresis, and quantity was determined using the PicoGreen DNA quantification assays (Life Technologies). Analysis of the DNA was performed using Illumina Infinium Methylation EPIC v1 BeadChips according to the manufacturer’s instructions. The methylation assays were carried out at the Roswell Park Cancer Institute Genomics Shared Resource. BeadChip-based methylation assays involve bisulfite conversion of the genomic DNA, in which unmethylated cytosines are converted to uracil. This is followed by primer extensions to query the percentage methylation at each of 862,927 (850 K) CpG dinucleotides, covering CpG islands (CGIs) within and around both promoter and non-promoter regions, as well as many non-island promoter regions, associated with 99% of RefSeq genes. The resulting dataset was processed with GenomeStudio (v2011.1) software using the Methylation Module (v1.9.0). After background correction and normalization to internal controls, the percentage methylation (AVG_Beta) at each CpG queried by the array was calculated. Poorly performing probes with missing values (AVG_Beta detection *p* > 0.05 occurring in more than one sample per subgroup), probes mapping to the X or Y chromosome, and SNP probes were removed.

### 2.9. Expression/Methylation Correlation Analysis

The methylation dataset was pre-processed from raw IDAT BeadArray files using the minfi v1.50.0 package [23] to generate a GenomicRatioSet object. Pre-processing steps included removal of probes mapping to X and Y chromosomes, SNPs, and multiple regions of the genome, which was followed by calculation of beta values. RNA-seq data were processed as described previously, with the exception that the hg19 genome build was used during alignment to facilitate integration with the methylation array annotation. Variance stabilizing transformed transcriptional expression values were annotated and packaged as a Biobase ExpressionSet object for the multiomics integration. Correlation analysis between methylation and transcriptomic data was performed using the correlationMethExprs() function in the MEAL (Methylation and Expression AnaLizer) v1.34.0 Bioconductor library in R [24]. For this, a conservative flanking region of 10,000 bp upstream and downstream of the CpG was used to define the expression-methylation probe pairs. Within this functional call, linear regression models were fit for each pair using the lm() function in the R stats library, and the resulting *p*-values were adjusted for multiple comparisons using p.adjust().

### 2.10. PCR

*RUNX1* mRNA was measured by qRT-PCR. cDNA was synthesized from 200 ng of RNA using the High-Capacity cDNA Reverse Transcription Kit (Cat No. 4368814; ThermoFisher). qPCR was performed using primers for *RUNX1* (Hs01021970_m1; ThermoFisher), the *YWHAZ* housekeeping gene (Hs03044281_g1), and the TaqMan™ Fast Advanced Master Mix for qPCR (Cat. No.: 4444556; ThermoFisher) on a QuantStudio 6 Flex Real-Time PCR unit (ThermoFisher). Changes in *RUNX1* expression were calculated using the 2^−DDCT^ methodology.

PCR array analysis of EMT-associated genes was performed as described previously [11]. Reverse transcription of RNA from EVT cells was carried out using the RT^2^ First Strand Kit (Qiagen). Transcribed cDNA was mixed with RT^2^ SYBR Green Mastermix (Qiagen), and equal quantities were placed in a 96-well RT^2^ Profiler PCR Array (Cat. #PAHS090Z, Qiagen) for 84 EMT-associated genes. The PCR Array was run on an ABI 7900HT Fast Block Real-Time PCR System (Agilent) using the recommended cycling conditions. A combination of five housekeeping genes (*ACTB*, *B2M*, *GAPDH*, *HPRT1*, *RPLP0*) was used to normalize the data. Statistical significance of the gene expression differences was determined from 2^−DDCT^ values using paired, multiple *t*-testing (Graphpad Prism 10.4.1).

### 2.11. Western Blotting

After washing with PBS, JEG3 cells were extracted into RIPA lysis buffer. Protein (30 µg/well) was loaded onto 8% SDS gels for electrophoresis. Proteins were transferred from the gels to a nitrocellulose membrane (Bio-Rad, Hercules, CA, USA), blocked (0.5% fat dried milk in PBS-Tween, 60 min), and incubated with an anti-RUNX1 polyclonal antibody (1:1000; GTX129100, Genetex, Irvine, CA, USA) or anti-ß-actin polyclonal antibody (1:1000; Cat# 4967S, Cell Signaling Technology, Danvers, MA, USA) at 4 °C overnight, followed, after washing × 3 with PBST, by a 60 min incubation with an anti-rabbit IgG coupled to HRP (1:1000; Cat# 4967S, Cell Signaling Technology). Blots were incubated with Pierce ECL Western Blotting Substrate (ThermoFisher) and visualized using a ChemiDoc MP imager (Bio-Rad). Blots were quantified using ImageJ (v1.64, NIH).

### 2.12. Statistical Analysis

Sex- and gestational-age-specific birthweight centiles were calculated according to Fenton and Kim [25]. Data from the qRT-PCR assays, Western blots, scratch, and invasion assays were checked for normality using the Shapiro–Wilk test. Statistical analysis was carried out using an unpaired, two-tailed *t*-test (Prism v10.4.1).

## 3. Results

### 3.1. Sample Characterization

**Figure 1:**
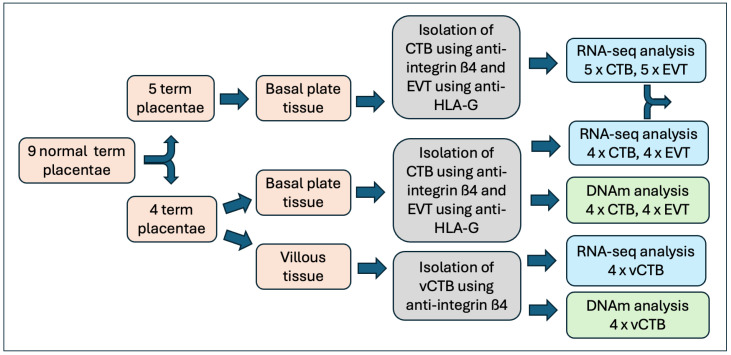
Flowchart showing preparative and analytical steps.

Placental tissue samples were obtained from nine normal term pregnancies (clinical data can be found in Table S1). CTB and EVT, obtained from the basal plate, were purified from all nine placentae and used for RNA sequencing. In four of the nine placentae, CTB from the chorionic villous core of the placenta (vCTB) was also obtained and analyzed. Illumina EPIC (800 K) DNA methylation assays were performed on vCTB, CTB, and EVT cell samples from the same subset of four samples (Figure 1). There were no differences in clinical parameters between the full set of samples and the EPIC subset.

For each placental sample, CTB and EVT were obtained from the same tissue and are thus paired. The vCTB extracted from chorionic villous tissue samples and the DNA utilized for the methylation assays were obtained from the same samples from which CTB and EVT were isolated for RNA preparation. Characterization of the vCTB, CTB, and EVT cells, measured as a percentage of cells staining for ITGß4-PE (vCTB, CTB) or HLA-G-PE (EVT), shows a mean purity of >95% for all three cell types. This is the same as data reported previously for these cells, which included assessments of cytokeratin-7, vimentin, HLA-G, integrin α1, and integrin α6 [11]. While ITGß4 is prominent only in CTB, there is a possibility that the antibody to HLA-G might pick up hematopoietic cells in the placenta; however, RNA-seq results (below) show similar levels of *CD45* (*PTPRC*) in vCTB, CTB, and EVT samples, all below the analytical cutoff (baseMean) used in analysis.

### 3.2. Comparison of CTB and EVT Transcriptomes Highlights Differential Expression of Genes Involved in Trophoblast Differentiation and EMP

**Figure 2:**
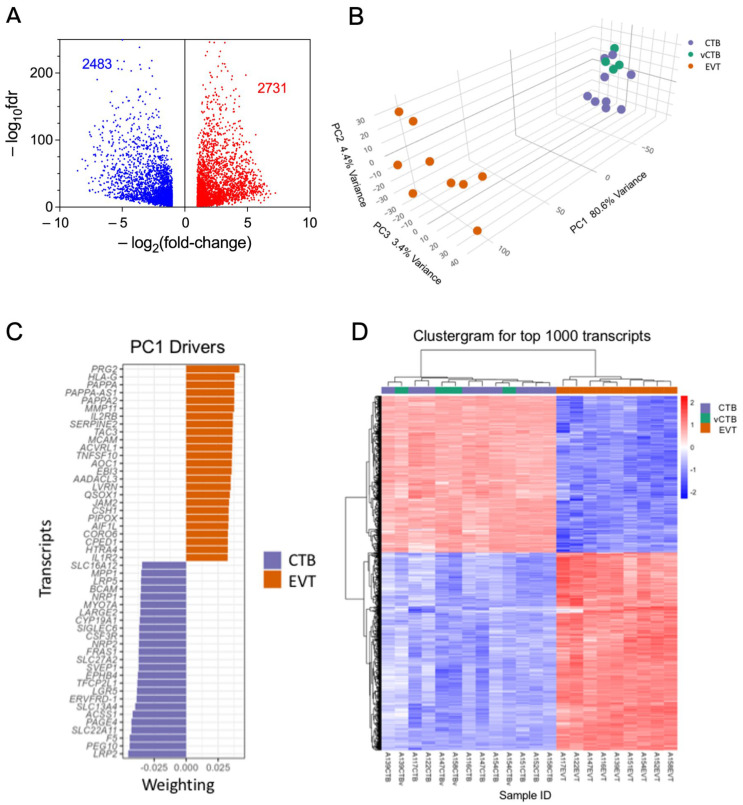
Analysis of RNA sequencing data. (**A**) Volcano plot of RNA sequencing DE data showing decreased expression in EVT (blue) and increased expression (red). Paired analysis, using the threshold parameters of (1) FDR ≤ 0.05, (2) featureCount (baseMean) ≥ 10, and (3) a fold change of ≥1.5. (**B**) PCA plot of CTB (purple), vCTB (green), and EVT (orange) using the full DE gene set. (**C**) Top 50 driver genes (25 positive, 25 negative) for the first component of the PCA. (**D**) Clustergram for CTB (purple), vCTB (green), and EVT (orange) for the top 1000 genes.

RNA sequencing of the nine sets of paired CTB and EVT yielded > 23,000 transcripts. Paired analysis, using the threshold parameters of (1) FDR threshold of ≤ 0.05, (2) featureCount (baseMean) ≥ 10, and (3) a fold change in expression of ≥2.0 restricted differential expression (DE) to 5214 transcripts. This comprised 2731 up-regulated genes and 2483 down-regulated genes in EVT compared to CTB (Figure 2A). Multidimensional visualization via principal components analysis of DE (fold change) between EVT, CTB, and vCTB (Figure 2B) shows that the CTB and EVT clusters are distinct and well separated. This confirms the expected clear discrimination between these cell types, based on both our flow cytometry results and gene expression profiling.

The vCTB cluster with the CTB, suggesting that the phenotypic differences between cytotrophoblast obtained from the basal plate (CTB) and villous core (vCTB) are small, relative to those between CTB and EVT. Investigation of sex as a variable, while desirable, revealed minimal differences; however, this is likely due to the small number of samples per group (four male, five female).

The marked separation in the first PCA component between CTB and EVT explains >80% of the variance. The top 50 leading-edge genes (25 with a positive coefficient, 25 with a negative coefficient) driving this separation are shown in Figure 2C. The clear difference between CTB and EVT is further confirmed by the clustergram for the top 1000 genes (Figure 2D), which also shows that the vCTB, while differentiated from the EVT, are intermixed with the CTB, indicating that the CTB obtained from the basal plate versus the villous core differ much less than either cell type from EVT.

### 3.3. EMP in Trophoblast Differentiation

Our results obtained previously from the PCR arrays of EMT-associated genes [10,11,26] showed an enrichment in EVT of genes promoting EMP. We extended these findings by investigating the correspondence between the RNA-seq results for the CTB/EVT differentiation process and a library of EMT genes assembled from multiple sources [27,28,29,30,31], comprising 564 genes. It should be noted that this library was assembled in large part from EMT-associated genes described in carcinomas, the most numerous source, many of which may not be active in trophoblast. In the intersection with our DE data, 336 genes overlapped with this EMT gene library. Among those (DE-EMT) genes are many regarded as canonical markers of the EMT, including cell junctional elements (*CLDN1*, *MARVELD3*, *OCLN*), cytoskeletal components (*ANK3*, *KRT19*), extracellular matrix genes (*COL5A1*, *FN1*, *ITGA5*, *ITGB4*), secreted proteases (*ADAM12*, *ADAM 19*, *MMP11*), and signaling genes (*BMP7*, *EGFR*, *TGFB2*). The high proportion of canonical pro-EMT genes showing up-regulated expression in EVT compared to CTB points to an EMT-like mechanism as the major contributor to EVT differentiation.

Perhaps most important is the response of regulatory transcription factors in differentiation. Several of the primary EMT master regulators are up-regulated in EVT, including *SNAI1* (6.7-fold), *TWIST2* (10.2-fold), and *ZEB1* (5.1-fold). Several other primary master regulators are significantly upregulated (*SNAI2*, *SNAI3*, *ZEB2*); however, their levels of expression fall below our analytical expression (baseMean) cut-off (Table S2). The exception is the reduced expression of *TWIST1* (−6.7-fold), which has been shown to promote CTB differentiation into syncytiotrophoblast [32]. Beyond the primary master regulators, there are multiple transcription factors, known to be associated with the EMT in other tissues [33,34], which also show significant changes indicative of an EMT-like mechanism.

In addition, there are a number of genes in the DE list that are expressed primarily in the placenta [35], including *CSH1*, *CSH2*, *PAPPA*, *GH2,* and *HLA-G*, genes that show major increases in EVT compared to CTB (Table S3). Also notable in that profile are the substantial decreases in trophoblast genes expressed primarily in the syncytial fusion process, including the retroviral genes, which generate the syncytial fusion proteins syncytin-1 and syncytin-2 (*ERVW-1*, *ERVFRD-1*).

### 3.4. Functional Enrichment of the EMP Mechanisms in Differentiation

Following the identification of genes differentially expressed between CTB and EVT, we determined the functional processes that are enriched among these DE genes. Data generated by DESeq2 was used to test for enrichment of the Hallmark gene sets (v7.4) obtained from the Molecular Signatures Database (MSigDB) collections (EVT vs. CTB; ≥2 fold change; baseMean > 10; FDR < 0.05). The extent (or loss) of enrichment for the top 15 processes is quantified in Table 1. This analysis revealed significant enrichment in biological processes associated with EMP.

**Figure 3:**
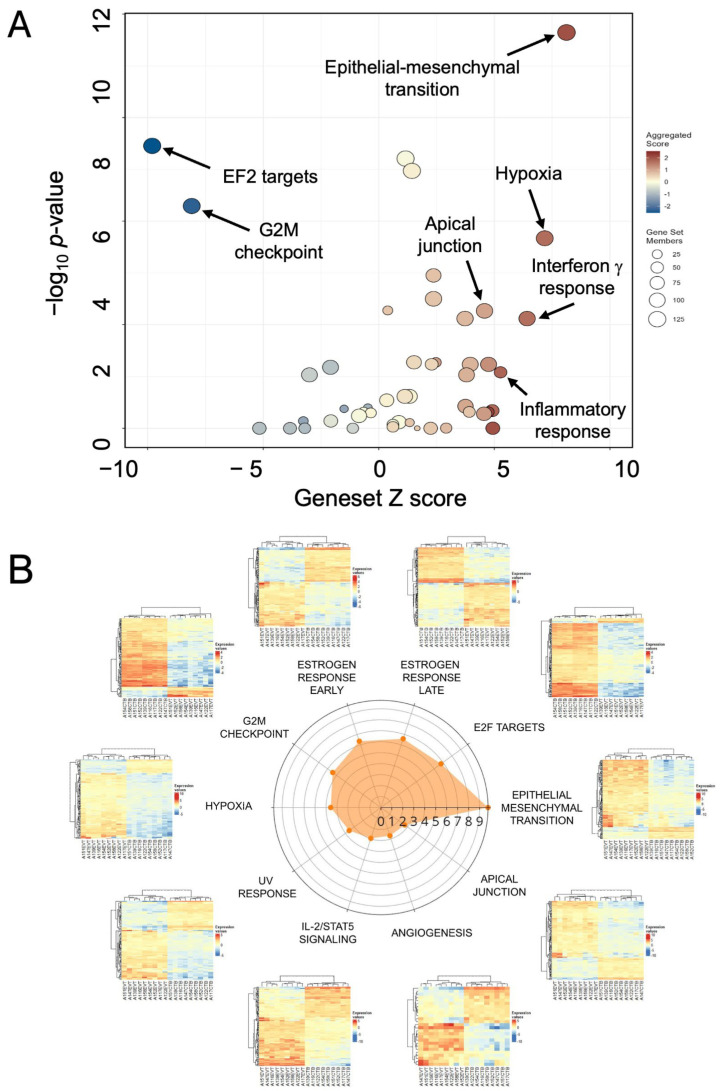
Functional enrichment of differentially expressed genes in EVT. (**A**) Volcano/bubble plot of enrichment drawn from the Hallmark database of gene sets. (**B**) Radar plot of the top 10 enriched gene sets. Each heatmap shows the nine lanes for CTB on the left and nine for EVT on the right.

The volcano-bubble plot in Figure 3A summarizes the functional enrichment results. It shows most conspicuously a significant enrichment in the EMT gene set, corroborating the role of this mechanism in CTB to EVT differentiation. These results are confirmed in the radar plot of enriched gene sets shown in Figure 3B. In parallel with up-regulation of the EMT gene set is a reduction in the G2M and E2F target gene sets, demonstrating a loss of proliferative capacity in the transformation from CTB to EVT.

### 3.5. Differences in EMP Genes Between Basal Plate and Villous Tree CTB

We investigated whether the changes seen in EVT compared to CTB might be a result of differences between the basal plate CTB (used as controls for the DE analysis) and the villous tree CTB (vCTB). We find that while the PCA and clustergram (Figure 2B,D) do not reveal obvious differences between CTB and vCTB, multiple genes show significant changes. Some 23 genes overlapping with the EMT library show differential expression;16 showing an increase in CTB compared to vCTB. Importantly, many of these genes show further changes in the transition to EVT; thus, there is a 5-fold increase in *ZEB1* between vCTB and CTB, with a further 5-fold increase in the transition to EVT (Table S4). These relatively limited changes suggest that while both cell types are on a differentiation continuum, the CTB shows a slight shift towards the mesenchymal compared to the vCTB.

### 3.6. Validation of the EMP and Designation of a Trophoblast EMP Signature

**Figure 4:**
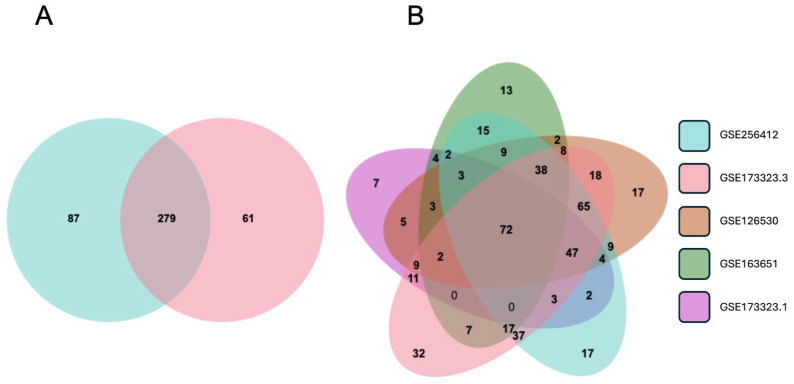
Differential gene expression between CTB and EVT in first- and third-trimester gene sets. (**A**) Venn diagram showing differentially expressed EMT-associated genes from third-trimester datasets GSE256412 (blue) and GSE173323 (red). (**B**) Venn diagram showing differentially expressed EMT-associated genes from third-trimester datasets GSE256412 (blue), GSE173323 (red), and first-trimester datasets GSE1265230 (brown), GSE163651 (green), and GSE173323 (purple).

For validation, we compared the differential expression of the 336 EMT-associated genes reported here with the third-trimester data in GSE173323 [36]. In the latter, there were 340 EMT-associated genes (out of our library of 564), providing an overlap of 279 with the current data (Figure 4A), supporting that an EMT-like mechanism is the primary process driving the differences between CTB and EVT. Furthermore, using the same library of EMT-associated genes, we analyzed the first-trimester CTB and EVT data reported in GSE126530 [37], GSE163651 [38], and GSE173323 [36] in combination with the third-trimester data reported here. The overlap between the five datasets contained 72 genes (Figure 4B, Table S5). This gene set provides a baseline trophoblast EMT signature applicable to both the first and third trimesters.

For the purpose of rapid determination of trophoblast status, we have identified an abbreviated set of highly expressed genes including the EMT marker genes *FN1*, *ITGA5*, *MMP11*, *ADAM19* (upregulated in EVT), *BMP7*, *MSX2*, *SERPINF1* (down-regulated in EVT), and placenta-associated genes displaying similar characteristics including *HLA-G* (up-regulated) and *ERVFRD-1* (down-regulated). We validated this set by calculating the fold change for each of these genes in three sets of first-trimester data (GSE126530, GSE163651, GSE173323) and two sets of third-trimester data (GSE173323, GSE256412/current). All sets showed similar changes (Table S6), although the third trimester changes were generally greater than those in the first trimester.

### 3.7. Is EVT Gene Expression Reversibly Modified by In Vivo Conditions?

One of the important questions, as yet unanswered, is the nature of the changes that slow EVT invasion and lead to the apparent regression from the more invasive, mesenchymal phenotype observed in the first trimester [10] to the less or non-invasive EVT found in the third trimester placenta [11]. It has been suggested that the changing nature of the environment, as EVT invades the decidua and myometrium, might be responsible for constraining the EVT invasion and reversing the mesenchymal shift. We sought to test this by comparing freshly isolated third-trimester EVT with the same EVT that had been cultured in vitro for 72 h. We reasoned that a period of 72 h would be sufficient to release the cells from most in vivo influences and potentially allow them to revert or relax to a phenotype unrestricted by decidual/myometrial cues. Following incubation, we assayed the paired EVT samples (freshly isolated EVT and 72 h-incubated EVT) using a PCR array of EMT-associated genes, as we have done previously [11]. Freshly isolated and incubated EVT were compared, using paired, multiple *t*-testing analysis (n = 3, FDR < 0.05, ≥2-fold change). Out of the 84 genes in the array, only one showed a significant difference; *OCLN* increased 2.2-fold in the in vitro incubated EVT compared to the freshly isolated EVT. This result is perhaps not surprising as isolated EVT attach to the plate and re-adhere to each other, a process involving junctional proteins such as occludin. These results are in contrast to data such as that reported by Zhou et al. for CTB [39], in which CTB from PE pregnancies demonstrated a significant degree of relaxation in gene expression over 48 h [39]. Our results indicate that the regressive EMP demonstrated in the shift from the invasive first-trimester EVT to the less invasive, more epithelial third-trimester EVT phenotype does not appear to be due to the sustained effect of a reversible environmental factor. While interaction or loss of interaction with an external agent might initiate the regression, the irreversible nature of the change seen here suggests a more permanent, potentially epigenetic form of regulation, supporting our motivation for the methylomic investigation.

### 3.8. Comparison of CTB and EVT Methylomes Highlights Widespread Losses and Focal Gains of CpG Methylation in EVT

**Figure 5:**
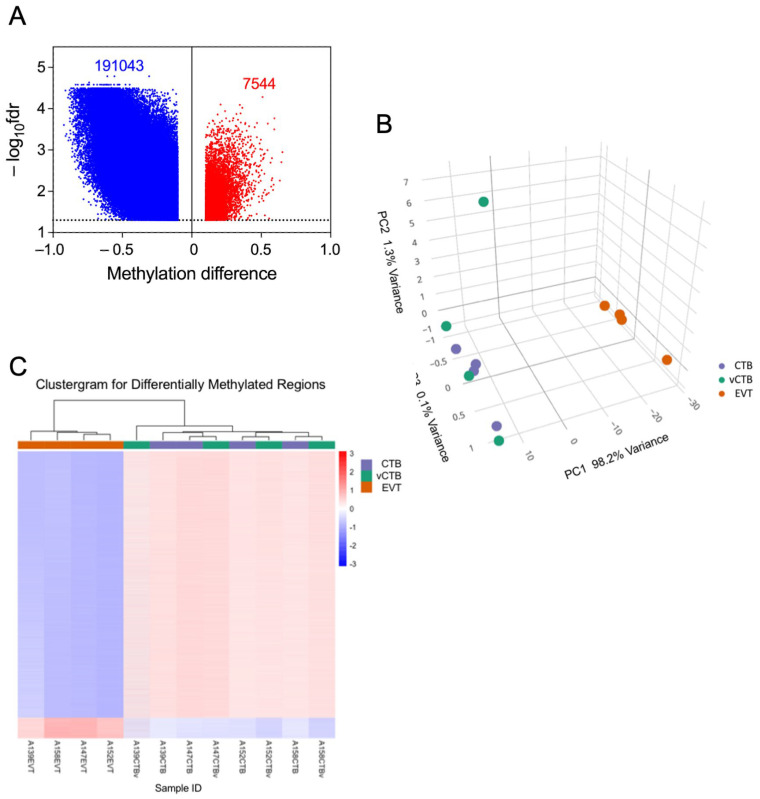
Differential methylation of genes in EVT compared to CTB. (**A**) Volcano plot showing differences in total methylation (decreased—blue, increased—red) between CTB and EVT. Dotted line marks fdr = 0.05 cut-off. (**B**) PCA plot of the methylome for CTB (purple), vCTB (green), and EVT (orange). (**C**) Clustergram showing differences in methylation between vCTB, CTB, and EVT.

Prior reports suggest that DNA methylation (DNAm) plays a role in regulating human trophoblast differentiation [40,41,42,43]. Because of the results in the previous section and these literature reports, we decided to investigate whether DNAm played a role in the differentiation process being investigated here. We analyzed DNA from EVT, CTB, and vCTB (n = 4, 4, 4) using the Illumina Methylation EPIC BeadChip array (~863 K CpGs). Of the ~23,000 genes covered in this assay, probes representing approximately 15,000 (~190 K CpGs on the BeadChip) demonstrated hypomethylation in EVT compared to CTB, as indicated in the volcano plot (Figure 5A). This shows the substantial asymmetry in the methylation changes between EVT and CTB, which is similar whether in gene promoter regions or elsewhere. The principal components analysis of the methylation data (Figure 5B) exhibits separate clustering of CTB/vCTB and EVT, similar to that shown for gene expression. There is an outlier in the CTB/vCTB set, a vCTB sample which is, however, well aligned with the other CTB samples in the PC1 dimension (accounting for 80% of the variance). Moreover, the clustergram for these samples (Figure 5C) shows clear separation between EVT and CTB/vCTB, without outliers. That hypomethylation dominates both cell types agrees with other studies showing the relative hypomethylation of the placenta or trophoblast compared to other somatic cells [43,44,45,46,47,48]. Strikingly, our data show that the EVT is hypomethylated to an even greater degree than CTB. Our RNA-seq data also shows that the *DNMT1*, *DNMT3A,* and *DNMT3B* methyltransferase genes, responsible for much of DNA methylation, were down-regulated 7.7-, 4.2-, and 3.8-fold, respectively, in the transition between CTB and EVT, perhaps explaining, in part, the broad hypomethylation observed in the EVT.

### 3.9. Correlation Between Gene Expression and Methylation

We examined the relationship between gene expression and methylation by testing the extent of correlation between the two analyses. Correlation was observed (FDR ≤ 0.05) for 5452 genes over >17,000 CpG sites. Intersection with our EMT gene library identified 188 EMT-associated genes showing correlation of expression with methylation (Table S7). Of these, 124 demonstrated a negative correlation, that is, decreasing expression with increasing methylation, while 54 showed a positive correlation. Analysis using the Hallmark gene sets revealed that the genes showing a negative correlation were clearly associated with the EMT and, to a lesser degree, with TGFß signaling.

### 3.10. Genes Showing Gain of Methylation, Differential Expression, and Involvement in EMP

In contrast to the genome-wide hypomethylation described above, a gain of methylation (GOM; ≥2 CpGs with ≥20% increase in methylation, FDR < 0.05) was associated with >1400 genes. We suggest that the gains of methylation, which occur in regulatory sequences including promoter, enhancer, and insulator regions, might be specific, directed events. Many of these GOM regions showed altered mRNA expression in the nearest genes. Meshing these GOM regions with our DE gene set revealed more than 700 genes that showed both gain of methylation and altered gene expression. Notably, the Hallmark EMT gene set was also enriched in this set of GOM-DE genes. The further intersection of these genes with those in the EMT library revealed a subset of 44 genes (GOM-DE-EMT genes; Table 2).

Among these genes, there were 15 where increased methylation was associated with increased gene expression and 29 where increased methylation was associated with decreased expression. We examined the role played by these genes in the EMT via reports in the literature and whether the changes in expression caused by increased methylation would have had the effect of promoting or inhibiting cellular progression from epithelial to mesenchymal across the EMT spectrum (Table S8). Those genes showing an increased expression as a result of increased methylation were generally associated with the promotion of the EMT. Those genes showing a decreased expression with increased methylation were generally associated with the reverse, promotion of the mesenchymal-epithelial transition (MET). A proportion in both categories was associated with both EMT and MET, depending on the tissue context.

### 3.11. A 1.3 Mb Domain Gain of Methylation in EVT Spans the EMT-Associated RUNX1 Gene

**Figure 6:**
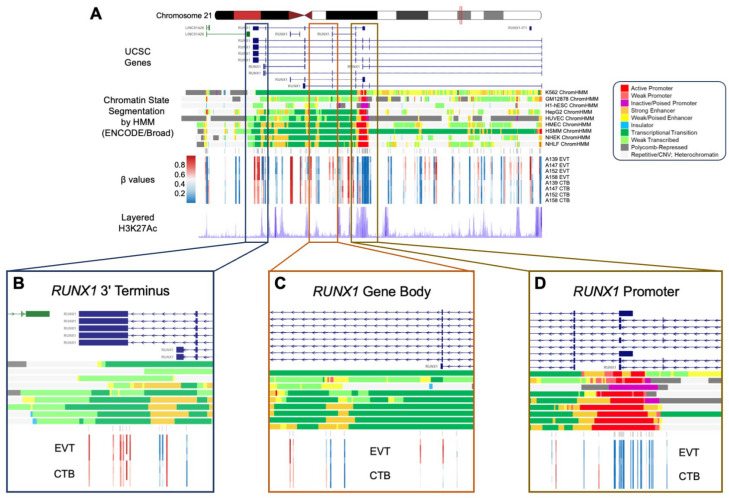
Regional RUNX1 gene body hypermethylation in EVTs relative to CTBs. (**A**) Integrative genomics viewer (IGV) visualization of the genomic region around RUNX1 (Chr21: 36,110,847–36,448,197; hg19 coordinates) displaying the chromosome 21 ideogram and the following tracks: (1) UCSC known genes track showing RUNX1 transcripts (blue) and antisense LINC01426 transcripts (green); (2) Chromatin State Segmentation by Hidden Markov Model (ChromHMM) track for nine ENCODE cell lines; (3) Illumina 850 k EPIC methylation array track showing positions of CpG sites being measured; (4) heatmap representation of DNA methylation (β values) for EVT and CTB samples; (5) layered H3K27Ac track (epigenetic mark for active regulatory elements). (**B**–**D**) Enlarged views of three regions from the RUNX1 gene (3′ terminus, gene body, 5′ promoter), showing DNA methylation changes in relation to genomic and epigenomic features. The ChromHMM display conventions are as in https://genome.ucsc.edu/cgi-bin/hgTrackUi?g=wgEncodeBroadHmm, accessed on 5 January 2019.

Among the genes showing GOM and increased expression is the transcription factor, *RUNX1*, a well-recognized participant in the EMT and a regulator of cell mobility [49,50,51,52]. A 1.3 Mb domain of extensive CpG hypermethylation on human chromosome 21 in EVT was observed to span the *RUNX1* gene. Importantly, this broad domain of GOM was interrupted by smaller regions of loss of methylation, with the GOM being excluded from the major *RUNX1* promoter element, which was relatively hypomethylated in EVT (Figure 6). *RUNX1* shows a high gain of methylation during the CTB to EVT differentiation process (GOM at 21 CpG sites) associated with a greater than 7-fold increase in mRNA expression (Table 2). The increased methylation in *RUNX1* is largely accounted for by hypermethylation of the gene body, a process which has been shown to be positively correlated previously with increased transcription [53] and confirmed in this report. Moreover, when compared to other canonical and non-canonical EMT transcription factors, *RUNX1* is only one of three that show a GOM and does so to a substantially greater degree than the other GOM-EMT transcription factors, *ASCL2* and *FOXK1* (Table S2), emphasizing its unique nature.

### 3.12. Knockdown of RUNX1 Leads to Altered Cell Migration and Invasion

**Figure 7:**
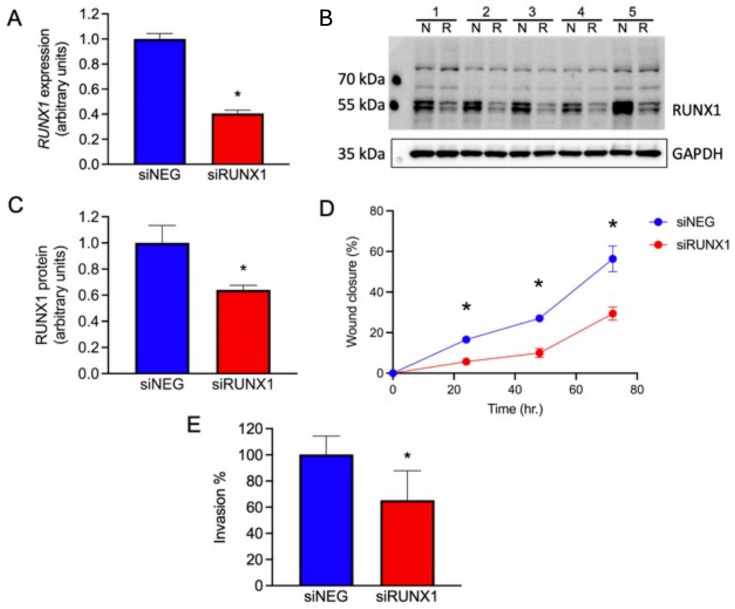
Role of RUNX1 in trophoblast. Changes in RUNX1 message, protein, and in migration and invasion of JEG3 cells following treatment with siNEG or siRUNX1. Statistical analysis was performed by a paired *t*-test for all measures. (**A**) RUNX1 gene expression, normalized to YWHAZ; n = 4, * *p* < 0.001. (**B**) Western blot of JEG3 cells for RUNX1 and GAPDH following treatment with siNEG (N) or siRUNX1 (R). (**C**) Quantification of RUNX1 protein expression, normalized to GAPDH, in JEG3 cells following treatment with siNEG or siRUNX1; n = 5, * *p* < 0.01. (**D**) Migration of JEG3 cells following treatment with siNEG or siRUNX1, measured as a percentage of wound closure; n = 3; * *p* < 0.05. (**E**) Invasion of siRUNX1-treated JEG3 through Matrigel-coated Transwell membranes, measured as a percentage of invasion by siNEG-treated cells; n = 16; * *p* < 0.001.

One potential role for the GOM-DE-EMT genes is stabilizing an EVT phenotype, leading to a restricted localization on the EMT spectrum. To test this concept functionally, we investigated the role of *RUNX1* as an example of a possible regulatory transcription factor. We treated JEG3, a trophoblast cell line derived from an invasive choriocarcinoma, with an siRNA against *RUNX1* and investigated the effect on *RUNX1* gene expression, RUNX1 protein expression, and JEG3 migratory capacity. As seen in Figure 7A, a 48-h treatment of JEG3 with siRUNX1 reduced gene expression by ~60% compared to treatment with the negative control (siNEG). Figure 7B shows a Western blot of siRUNX1-treated JEG3 cells using a monoclonal anti-RUNX1 antibody. The quantification of the Western blot is displayed in Figure 7C, which shows a ~55% reduction in RUNX1 protein in the siRUNX1-treated cells compared to siNEG-treated cells. After demonstrating the knockdown of the *RUNX1* gene and RUNX1 protein, we measured the migratory capacity of siRUNX1-treated JEG3 cells using a scratch assay (Figure S1). Figure 7D shows that the migratory capacity of the siRUNX1-treated cells was reduced by ~60% compared to the siNEG-treated controls at 72 h, paralleling the knockdown in mRNA and protein. Figure 7E shows that knockdown of *RUNX1* mRNA and protein leads to reduced invasion through Matrigel, a different process than migration, and consistent with the known role of *RUNX1* as a pro-EMT gene in other systems. This is consistent with in vivo information and the in vitro data described above.

## 4. Discussion

Our transcriptomic and methylomic comparison between term EVT and CTB has revealed substantial differences. In the transcriptomic comparison, we found over 5200 genes displaying gene expression changes. When this list was compared with a library of genes drawn from a broad set of EMT databases, there was a significant overlap between EMT genes and those showing altered expression in the EVT/CTB comparison. Most of the EMT master regulatory transcription factors and other EMT-associated transcription factors showed altered expression indicative of EMP. This solidly supports the operation of an EMP mechanism in trophoblast differentiation. This is substantiated by the finding of the Hallmark EMT gene set as the most prominently enriched functional process. Notably, the altered expression of EMT-associated genes in third-trimester EVT was not diminished by in vitro incubation, indicating that the differential expression was not reversible by separation from the in vivo environment.

Examining trophoblast methylation status revealed a substantial degree of hypomethylation in the EVT, even compared to the already-hypomethylated CTB. However, a small fraction of the sites tested demonstrated a gain of methylation. The intersection between these gains of methylation genes and the trophoblast EMT genes generated a group with both gains of methylation and differential expression. Functional testing of one of the primary genes in this group, *RUNX1*, confirmed its participation in the migration and invasion processes characteristic of EVT, supporting that a small number of genes showing gain of methylation in EVT play a regulatory role in the trophoblast differentiation process.

### 4.1. Confirmation of EMP in Trophoblast Differentiation

The substantial overlap of DE genes with the library of EMT genes demonstrates that an EMT-like mechanism is responsible, in large part, for the differentiation changes we observe. Our assessment aligns with the consensus guidelines from the EMT International Association [54]. The well-described changes in cellular properties known to occur during an EMT and observed in CTB-to-EVT differentiation are associated with molecular changes observed with EMP and stimulated by core transcription factors [10,55]. These are supplemented by our previous observations showing changes in key EMT-associated proteins, including occludin, E-Cadherin, fibronectin, matrix metalloproteinase-2, integrin a1, and integrin a6 [11].

Confirmation of an EMP mechanism raises the question of the location of third-trimester EVT on the EMT spectrum. While first-trimester EVT appears to be well-advanced towards the mesenchymal limit of the EMT spectrum [10], third-trimester EVT appears to have undergone regression back towards the epithelial pole [11], a sign of the EMP, which characterizes trophoblast differentiation. First- and third-trimester EVT have been obtained from similar locations in both cases, ruling out changes ascribed to different EVT subtypes. Thus, the differential gene expression we observe in term placental EVT appears to be the result of at least two major forces. The first is the initial drive towards differentiation, which results in the mesenchymal phenotype of first-trimester EVT. The second force is that which reduces and/or reverses the EVT progression, leading to the loss of invasiveness, loss of proliferative capacity, and regression of EVT on the EMT spectrum. The data obtained here is, in all probability, an echo of the differentiation process which takes place early in gestation, blunted by the subsequent regression.

### 4.2. Changes in the DNA Methylation Profile

Several aspects of the changes in the DNA methylation profile between CTB and EVT stand out. The first is the overwhelming degree of hypomethylation in the EVT cells. That hypomethylation dominates both cell types agrees with other studies showing the relative hypomethylation of the placenta or trophoblast compared to other somatic cells [43,44,45,46,47,48]. The interesting comparison here is to the aberrant DNA methylation (i.e., genome-wide hypomethylation) observed in cancer cells [56]. Within cancer cells, DNA methylation levels are reduced in regions of low CpG density compared with normal cells, while a subset of CpG islands are hypermethylated in a cell-specific manner, mainly targeting CpG islands in gene expression regulatory elements. This frequently results in alterations to genes controlling cell adhesion, migration, and invasion [56], properties shared with EVT. These pathways seem to be associated with the EMT-related gene expression changes that correlate with methylation. The similarity to metastasizing cancer cells, which employ EMT as a transforming mechanism, again supports the role of EMP in trophoblast differentiation.

The cause of the hypomethylation in the EVT is unknown. However, a possible contributing factor is the reduction in DNA methyltransferase expression, as both *DNMT3A* and *DNMT3B* show a gain of methylation within their promoter regions, while *DNMT1* shows a gain of methylation in other regulatory regions. Our transcriptomic results also show a major decrease in *UHRF1* (−17.1-fold), which codes for a ubiquitination factor crucial for methylation. *UHRF1* depletion results in significant promoter demethylation and gene upregulation in cancer cells [56]. However, this, like the actions of *TET1* and *TET2,* which are normally associated with demethylation, requires the DNA replication-dependent dilution of DNA methylation marks by the inactivation of de novo DNA methylation. As third-trimester CTB and EVT are relatively non-proliferative, this indicates that demethylation processes observed in EVT may well occur earlier in gestation, at a time when these cells still retain proliferative capacity. These data would support the generation, early in gestation, of a mobile, invasive cell phenotype for the EVT, similar to cancer cell metastasis, followed later by a reduction in invasive capability, occurring at a time when these cells are still proliferative and generating the hypomethylated EVT we observe in the third trimester. The timing of the decline in proliferative capacity is as yet unclear, but it marks the demarcation point for DNA replication-dependent demethylation events. The methylation changes in CTB between early and late first trimester observed by Nordor et al. [42] may mark the time at which changes resulting in abnormal invasion occur.

### 4.3. Gain of Methylation and Trophoblast EMP

By contrast with the generalized hypomethylation, EVT also shows a gain of methylation in a small proportion of the sites surveyed. The very limited extent of the gain of methylation, in the face of overwhelming EVT hypomethylation and the decreases in the expression of the methyltransferases, suggests to us that these events may be specifically directed, potentially with functional consequences. Within the DE gene set, a fraction shows GOM and are identifiable as associated with an EMT (Table 2). An examination of these 44 genes reveals a mixture of pro-EMT and pro-MET (mesenchymal-epithelial transition) gene expression changes. For some genes, such as *TGFß1*, *FLT4,* and *MICALL2*, an increase in expression may be associated with mechanisms promoting a pro-EMT shift, towards the mesenchymal phenotype. Others with increasing expression, such as *COL5A1*, are thought to be consequences of that shift rather than mediators, although they do form part of the mechano-transduction processes which accompany the EMT [57,58]. Most of the genes showing increased expression associated with a gain of methylation support a pro-EMT shift across the EMT spectrum. By contrast, for methylated, EMT-associated genes showing a decrease in expression, the primary effects appear to support a pro-MET role. Overall, these two gene sets demonstrate features that both promote and suppress the trophoblast EMT (Table S8).

### 4.4. Functional Effects of Methylation

Despite the specific gains in methylation and an association with EMT, the consequences of these changes have not been tested functionally in trophoblasts. Therefore, we chose to investigate the role of *RUNX1*, as an example of an EMT-associated gene for which the GOM is positively correlated with expression in EVT. In the human placenta, RUNX1 protein was observed in chorionic villi and decidua by Bermudez et al. [59], but in the absence of co-labeling, cell type localization could not be confirmed. By contrast, the findings of Ponder et al. point specifically to the presence of RUNX1 protein in cell column CTB and in EVT throughout gestation [60]. *RUNX1* has also been shown to play a crucial role in the differentiation of human embryonic stem cells (HESCs) to early mesodermal lineages. In these experiments, Van Oudenhove et al. discovered that depletion of *RUNX1* not only impaired the EMT but also caused a loss in cellular motility, as reflected in a scratch assay [52]. Our findings in JEG3 trophoblast lineage cells replicate this finding and further demonstrate that loss of *RUNX1* mRNA and its encoded protein decreases trophoblast invasion, consistent with the general pro-EMT function of this gene. Our findings of increased *RUNX1* gene body methylation and upregulation of its mRNA in EVT, combined with the effects of its silencing on JEG3 cell migration, suggest that it plays a significant role in trophoblast differentiation. Another gene, *ASCL2*, which shows an increase in methylation paralleled by an increase in expression, displays a similar pattern, in that disruption of expression leads to an impairment of differentiation [61].

### 4.5. Consequences of Methylation and Gene Expression Changes

We interpret our findings in the following manner. Trophoblast EMP is controlled by the balance of forces advancing cells towards either the epithelial or mesenchymal ends of the EMT spectrum. Changing the expression of genes controlling specific aspects of EMP will drive cells one way or the other. Thus, in the first trimester, there is high expression of genes such as *ZEB2*, which promote transition towards the invasive, mesenchymal cell type [10]. As gestation progresses, other gene expression changes moderate this drive such that the cells lose invasiveness but remain balanced between the more extreme epithelial and mesenchymal phenotypes.

We believe that the specific gains of methylation we observed may be part of a balancing act, preventing cells from regressing entirely to the epithelial phenotype while losing the invasive ability characteristic of the more mesenchymal, first-trimester EVT. These gains in methylation lock cells displaying EMP into a non-proliferative, non-invasive phenotype. Supporting this contention is data from Horii et al., who show that the differences in methylation between primed and naïve induced pluripotent stem cell (iPSC)—derived EVT appear to have significant consequences for invasiveness [62]. What remains unknown at this point are those factors, acting through the (de)methylation pathways, which initiate and regulate the equilibrium of the third-trimester EVT. The absence of the decidua in tubal pregnancies, where invasion is not arrested, is very instructive in this regard [63,64], suggesting that external signals, such as those produced by the decidua, may play a major role in the initial arrest of invasion.

### 4.6. Study Limitations

The number of samples analyzed in this type of study is always a limitation, although we believe that the pairing of CTB and EVT derived from the same tissue may have compensated in part for that particular shortcoming. As with many studies of this type, the conclusions reached here are limited by the fact that we used only third-trimester cells in this analysis. It is important to note, however, that this study was designed to act as a baseline for third-trimester investigations of pathological pregnancy conditions and has, thus, fulfilled its design expectations. Another limitation lies with the assessment of EMT genes. While we have used available libraries and databases, they are limited in their coverage and composed primarily of genes identified in cancer studies. There are many genes that, while identified as being associated with the EMT in the literature, are not in our library, and, conversely, many genes in the library may not be involved in trophoblast EMP.

We limited our functional testing to *RUNX1* as a good example of a gene that shows gain of methylation. The substantial increases in methylation and expression meant we were not examining a marginal event, while its role as a transcription factor underlines its importance. Further testing the individual roles of GOM-DE-EMT genes and the collective effects on EMP will require a significant degree of gene manipulation in realistic trophoblast models such as differentiating trophoblast stem cells and, as such, is beyond the scope of this study. In our functional testing, we also limited the cell type to JEG3 choriocarcinoma cells. While these are not EVT cells, they are proliferative and invasive trophoblast cells expressing HLA-G, which provide a useful model in which to test the role of *RUNX1*. Similarly, we used only one type of siRNA to knockdown *RUNX1*. While we are aware of the potential for off-target effects when unverified by a different siRNA, the fact that we were able to knock down RUNX1 protein by ~60% and to see reductions in both migration and invasion by the siRNA-treated cells convinced us that the *RUNX1* is involved in the regulation of a crucial aspect of trophoblast EMP.

The EVT analyzed in this report was isolated from the placental basal plate. It is possible that they may differ to some degree from those found in the deep decidua or upper layer of the myometrium. They are, nevertheless, invasive cells which have, in large part, moved beyond the villous tips and into the decidua, similar to the first trimester EVT, which were not obtained from the placental bed [36]. The information on first- and third-trimester DE in the latter report shows great similarity to our third-trimester data. Nevertheless, both investigations have skirted another limitation, which has become increasingly apparent from single-cell RNA-seq studies: the possible presence of cells regarded as intermediate between CTB and EVT [65,66,67]. The differences between vCTB and CTB provide support for the existence of this continuum of cell types; however, the numbers, temporal characteristics, and transience of these intermediates are a continuing issue.

## 5. Conclusions

Our gene expression results confirm that the differentiation of CTB into EVT includes the substantial involvement of an EMT-like mechanism. The differential DNA methylation profile demonstrates that the differentiation event is accompanied largely by EVT hypomethylation. The exception is the small number of genes showing a gain of methylation, many of which are associated with changes in gene expression. Within this latter group are genes that participate in EMP, one example being *RUNX1*, which shows a gain of gene body CpG methylation. We demonstrated that *RUNX1* likely plays a significant role in the behavior of trophoblast cells, illustrated by changes in migratory rate and invasive efficiency upon *RUNX1* depletion. The GOM-DE-EMT genes show changes in expression that promote both EMT and MET. This leads us to propose that the change in the methylation profile is one means by which movement of these third-trimester cells on the EMT spectrum is constrained, locking them into a stable configuration which is non-invasive but phenotypically mesenchymal.

## Figures and Tables

**Table 1:** Functional enrichment of biological processes in EVT compared to CTB.

Biological Process	Z Score	Adj. *p* Value
HALLMARK_EPITHELIAL_MESENCHYMAL_TRANSITION	8.12	1.71 × 10^−10^
HALLMARK_HYPOXIA	7.18	2.63 × 10^−5^
HALLMARK_INTERFERON_GAMMA_RESPONSE	6.42	2.77 × 10^−3^
HALLMARK_INFLAMMATORY_RESPONSE	4.75	4.15 × 10^−2^
HALLMARK_APICAL_JUNCTION	4.58	1.99 × 10^−3^
HALLMARK_TNFA_SIGNALING_VIA_NFKB	3.96	4.15 × 10^−2^
HALLMARK_GLYCOLYSIS	3.74	2.77 × 10^−3^
HALLMARK_TGF_BETA_SIGNALING	2.47	4.15 × 10^−2^
HALLMARK_IL2_STAT5_SIGNALING	2.37	1.11 × 10^−3^
HALLMARK_UV_RESPONSE_DN	2.37	2.68 × 10^−4^
HALLMARK_ANDROGEN_RESPONSE	2.29	4.15 × 10^−2^
HALLMARK_APOPTOSIS	1.53	4.15 × 10^−2^
HALLMARK_ESTROGEN_RESPONSE_EARLY	1.44	4.38 × 10^−7^
HALLMARK_MITOTIC_SPINDLE	−2.09	4.76 × 10^−2^
HALLMARK_G2M_CHECKPOINT	−8.09	3.66 × 10^−6^

**Table 2:** Differentially expressed genes associated with EMT, which show a gain of methylation.

Gene ID	Fold Change	CpG Gain	Gene ID	Fold Change	CpG Gain	Gene ID	Fold Change	CpG Gain
*HSPG2*	38.4	3	*PTPN14*	−2.4	3	*FGFR2*	−19.1	9
*ASCL2*	37.3	3	*NOTCH1*	−3.0	2	*BMP7*	−24.0	5
*LAIR2*	31.7	2	*TPD52L1*	−2.9	2	*MSX2*	−46.8	2
*PMEPA1*	27.0	2	*MAP3K4*	−3.0	5	*LRP5*	−91.4	6
*TGFB1*	17.5	3	*TGM2*	−3.1	2	*SVEP1*	−93.6	2
*FLT4*	13.9	8	*SYK*	−3.7	2	*ZBTB7C*	−104.2	6
*MICALL2*	12.5	12	*IGF1R*	−4.2	2	*LARGE2*	−114.9	2
*LPCAT1*	10.2	2	*FOXK1*	−4.8	2	*NRP2*	−123.0	2
*TWIST2*	10.2	2	*BCL2*	−6.9	2	*FRAS1*	−128.1	2
*COL5A1*	9.9	47	*MET*	−7.73	2	*EPHB4*	−140.9	5
*RUNX1*	7.5	21	*FBN2*	−12.3	2	*ACSS1*	−189.9	7
*FSCN1*	6.2	7	*FXYD3*	−13.1	3	*PEG10*	−195.8	5
*COL4A2*	4.4	4	*SPINT1*	−14.3	3	*SLC27A2*	−248.2	3
*BMP1*	3.8	2	*ALDH1A3*	−14.8	2	*LRP2*	−293.8	2
*PLOD1*	3.5	2	*JAG1*	−15.8	2			

## Data Availability

The RNA-seq and DNA methylation data can be found in the NCBI GEO database under the accession numbers GSE256412 and GSE259304, respectively.

## Supplementary Materials

The following supporting information can be downloaded at: https://www.mdpi.com/article/10.3390/cells14130970/s1, Table S1: Demographic characteristics of pregnancies from which placental tissue was obtained; Table S2: Differential expression and loss/gain of methylation in EMT master regulator or other EMT-associated transcription factors; Table S3: Differential expression of placental genes between CTB and EV; Table S4: EMT-associated genes showing differential expression in the CTB/vCTB and EVT/CTB comparisons; Table S5: Core trophoblast EMP gene differential expression signature; Table S6: Validation of an abbreviated EMP gene signature; Table S7: EMT-associated genes showing correlation between methylation and differential expression; Table S8: Potential effects on EMP of altered expression of DE-EMT genes that show a gain of methylation. Figure S1: Scratch assay images. Images show, from left to right, JEG3 cell migration following a scratch made by a 1 mm pipette tip. The upper set show migration of JEG3 cells treated previously with the siNEG control and the lower set show migration of cells previously treated with siRUNX1, as detailed in Materials and Methods, Section 2.3.
