# Supplementary material for: Epigenetic Changes Regulating Epithelial–Mesenchymal Plasticity in Human Trophoblast Differentiation
Source: Cells. 2025 Jun 24;14(13):970. doi: 10.3390/cells14130970 (PMC12249213; doi:10.3390/cells14130970)
Supplement: Supplementary file 1 [file cells-14-00970-s001.zip › cells-3668026-supplementary/Figure S1.pdf]

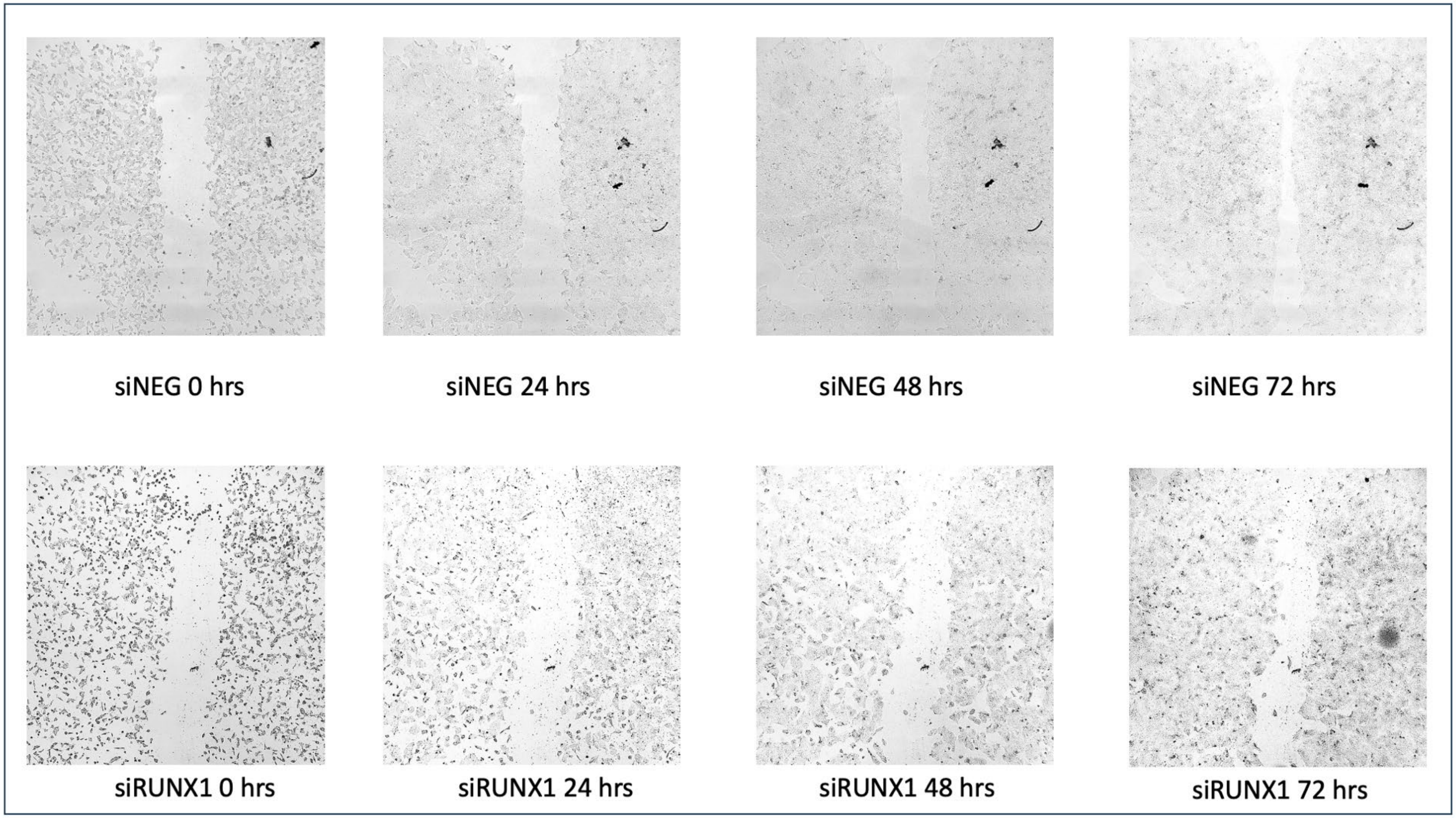

Figure S1: ***Scratch assay images.*** *Images show, from left to right, JEG3 cell migration following a scratch made by a 1 mm pipette tip. The upper set show migration of JEG3 cells treated previously with the siNEG control and the lower set show migration of cells previously treated with siRUNX1, as detailed in Materials and Methods, section 2.3*
