# Supplementary material for: Epigenetic Changes Regulating Epithelial–Mesenchymal Plasticity in Human Trophoblast Differentiation
Source: Cells. 2025 Jun 24;14(13):970. doi: 10.3390/cells14130970 (PMC12249213; doi:10.3390/cells14130970)
Supplement: Supplementary file 1 [file cells-14-00970-s001.zip › cells-3668026-supplementary/Table_S1.pdf]

Supplementary Table S1: Demographic characteristics of pregnancies from which placental tissue was obtained

**Parameter**	**Full sample set (n=9)**	**EPIC set (n=4)**
Maternal age (yrs)	32.8 ± 1.2	33.5 ± 1.8
Pre-pregnancy BMI	25.8 ± 1.7	24.8 ± 2.6
Gestational age at delivery (wks)	38.9 ± 0.1	39 ± 01
Fetal sex	M4 / F5	M2 / F2
Birthweight (g)	3383 ± 92	3295 ± 58
Birthweight centile	56.8 ± 7.2	49.5 ± 4.1
Placental weight (g)	514 ± 17	483 ± 14
