# Supplementary material for: Epigenetic Changes Regulating Epithelial–Mesenchymal Plasticity in Human Trophoblast Differentiation
Source: Cells. 2025 Jun 24;14(13):970. doi: 10.3390/cells14130970 (PMC12249213; doi:10.3390/cells14130970)
Supplement: Supplementary file 1 [file cells-14-00970-s001.zip › cells-3668026-supplementary/Table_S2.pdf]

Supplementary Table S2: Methylation differences in EMT master regulator or other EMT-associated transcription factors

Transcription factor type	Name	Fold change in gene expression	Methylation difference (number of sites)	
			**Loss of DNAm**	**Gain of DNAm**
**EMT master regulator TF**	SNAI1	6.72	4	
(Canonical EMT TF)	SNAI2	1.96		
	SNAI3	2.79		
	TWIST1	-6.67		
	TWIST2	10.17		1
	ZEB1	5.08	11	
	ZEB2	3.76	13	
**Non-canonical EMT TF**	ALX1	3.33		
	ASCL2	37.31		3
	BACH1	2.08	11	
	FOSL2	2.43	3	
	FOXC1	-12.5		
	FOXF2	31.01		
	FOXK1	-4.76	7	1
	FOXM1	-14.29		
	FOXN2	5.75	2	
	FOXQ1	8.12		
	GATA6	13.27	4	
	JUNB	1.63		
	KLF8	3.74		
	KLF10	2.31	1	
	OVOL1	-2.70	2	
	OVOL2	3.38	2	
	PRRX1	8.34	6	
	RUNX1	7.54	12	21
	SIX1	-12.5	1	
	SOX4	3.34	1	
	TBX3	-12.5		
	TEAD1	4.94	56	
	TEAD2	25.04		
	TEAD4	-9.09		
	WT1	2.20	8	
	ZBTB38	1.63	22	
