# Supplementary material for: Epigenetic Changes Regulating Epithelial–Mesenchymal Plasticity in Human Trophoblast Differentiation
Source: Cells. 2025 Jun 24;14(13):970. doi: 10.3390/cells14130970 (PMC12249213; doi:10.3390/cells14130970)
Supplement: Supplementary file 1 [file cells-14-00970-s001.zip › cells-3668026-supplementary/Table_S3.pdf]

Supplementary Table S3: Differential expression of placental genes between CTB and EVT.

Gene ID	Fold Change
CSH2	38.00
CSH1	37.41
CSHL1	25.91
PAPPA	22.96
HLA-G	21.39
PAPPA2	18.63
PLAC8	15.98
GH2	9.65
LEP	9.00
PSG11	7.77
PSG5	6.42
HSD3B1	5.67
PSG8	5.41
PTN	4.46
PSG3	3.91
PSG7	2.63
PSG1	2.40
PSG9	2.29
PLAC1	2.24
PSG4	2.22
EDNRB	2.14
PLAC9	2.02
MID1	1.74
PLAC2	-1.87
PLAC4	-6.15
ERVW-1	-14.24
ERVV-2	-19.41
ERVV-1	-22.80
CYP19A1	-164.72
SIGLEC6	-213.61
PEG10	-329.45
INSL4	-332.75
ERVFRD-1	-343.84
