# Supplementary material for: Epigenetic Changes Regulating Epithelial–Mesenchymal Plasticity in Human Trophoblast Differentiation
Source: Cells. 2025 Jun 24;14(13):970. doi: 10.3390/cells14130970 (PMC12249213; doi:10.3390/cells14130970)
Supplement: Supplementary file 1 [file cells-14-00970-s001.zip › cells-3668026-supplementary/Table_S4.pdf]

Supplementary Table S4: EMT-associated genes showing differential expression in the CTB/vCTB and EVT/CTB comparisons

Gene ID	Fold Change CTB/vCTB	Fold Change EVT/CTB
LUM	8.22	5.76
DCN	7.68	5.65
CYP1B1	7.56	3.54
CDH11	7.30	5.64
VIM	5.75	4.01
COL6A3	5.73	6.77
ZEB1	5.45	5.08
VCAN	5.32	6.8
COL6A2	3.93	14.27
TIMP1	3.75	27.67
COL3A1	2.98	4.48
LGALS1	2.70	13.66
SDC2	2.58	17.77
IGFBP4	2.26	5.35
TXNIP	2.08	4.38
S100A4	2.01	13.9
ZYX	-1.88	0
GSK3A	-1.98	0
TCF7	-2.00	0
LAMA5	-2.05	-2.22
GATA3	-2.46	2.76
DLX4	-2.52	3.06
EGR1	-2.86	0
NFIC	-3.30	0
MUC16	-3.69	0
