# Supplementary material for: Epigenetic Changes Regulating Epithelial–Mesenchymal Plasticity in Human Trophoblast Differentiation
Source: Cells. 2025 Jun 24;14(13):970. doi: 10.3390/cells14130970 (PMC12249213; doi:10.3390/cells14130970)
Supplement: Supplementary file 1 [file cells-14-00970-s001.zip › cells-3668026-supplementary/Table_S5.pdf]

Supplementary Table S5: Core trophoblast EMP gene differential expression signature.

**Gene ID**	**Gene ID**	**Gene ID**
ADAM19	FXYD3	MT1E
ALDH1A3	GLT8D2	MTDH
ANK3	HPGD	MTUS1
AURKA	IGFBP3	NUAK1
B3GNT7	IL1R1	OCLN
BCL2	ITGA5	PARP1
BMP7	ITGA6	PTN
CA2	ITGB1	RGS2
CDR2	KLF6	RGS3
CDS1	KRT19	RUSC2
DAB2	LAMA1	SERPINF1
EGFR	LHX2	SLC27A2
ELF5	LIMA1	SMAD3
EML1	LIMS2	SPINT1
ENG	LOXL1	ST14
EZH2	LOXL2	STAT5B
FAM163A	MAP3K4	TBX3
FAM169A	MAPK14	TGFB2
FGFR1	MARVELD3	TGM2
FGFR2	MCAM	TMEFF1
FLT4	MET	TNF
FN1	MFAP5	TPM1
FST	MME	YAP1
FSTL1	MSX2	ZNF165
