# Supplementary material for: Epigenetic Changes Regulating Epithelial–Mesenchymal Plasticity in Human Trophoblast Differentiation
Source: Cells. 2025 Jun 24;14(13):970. doi: 10.3390/cells14130970 (PMC12249213; doi:10.3390/cells14130970)
Supplement: Supplementary file 1 [file cells-14-00970-s001.zip › cells-3668026-supplementary/Table_S6.pdf]

Supplementary Table S6: Validation of an abbreviated EMP gene signature

Gene symbol	GSE126530	GSE163651	GSE173323	GSE173323	This report
	Fold change - first trimester			Fold change - third trimester	
BMP7	-6.6	-24.6	-8.6	-23.1	-23.3
ERVFRD-1	-10.5	-34.8	-24.3	-38.2	-166.7
MSX2	-5.7	-12.9	-14.5	-31.6	-45.5
SERPINF1	-4.9	-19.5	-9.3	-37.7	-40.0
ADAM19	9.5	10.2	29.1	69.6	41.1
FN1	9.8	14.7	18.7	131.0	32.9
HLA-G	10.3	10.1	24.0	417.0	56.0
ITGA5	9.5	8.5	17.3	96.3	29.9
