# Supplementary material for: Epigenetic Changes Regulating Epithelial–Mesenchymal Plasticity in Human Trophoblast Differentiation
Source: Cells. 2025 Jun 24;14(13):970. doi: 10.3390/cells14130970 (PMC12249213; doi:10.3390/cells14130970)
Supplement: Supplementary file 1 [file cells-14-00970-s001.zip › cells-3668026-supplementary/Table_S7.pdf]

Supplementary Table S7: EMT-associated genes showing correlation between methylation and differential expression

**Positive correlation**		**Negative correlation**				**Conflict**
**Gene ID**		**Gene ID**				**Gene ID**
ADAM12	*MBP*	*ADAM19*	*GJB3*	*MCAM*	*SDC2*	*ADAM12*
ANK3	*MET*	*AXIN1*	*GLRX*	*MFAP5*	*SEMA4C*	*FXYD3*
AURKA	*MICALL2*	*B3GNT7*	*GSN*	*MMP11*	*SERPINE1*	*HRAS*
BCL2	*MTUS1*	*BMP1*	*HIF1A*	*MMP14*	*SERPINE2*	*IL1B*
BRAF	*NRP2*	*CAMK2N1*	*HOXB7*	*MMP2*	*SLC16A3*	*LPCAT1*
CDKN1B	*OCLN*	*CAPN2*	*HPGD*	*MTDH*	*SLC39A6*	*MSX2*
CDKN2A	*PLAU*	*CAV1*	*HSPB1*	*MUC1*	*SLPI*	*PARP1*
CDKN2C	*PLXNB1*	*CAV2*	*ID1*	*MYCN*	*SMAD3*	*SLC27A2*
CLDN1	*PPL*	*CDR2*	*IFI30*	*NDRG1*	*SMAD7*	*SYK*
COL8A1	*PROM1*	*CLU*	*IGFBP3*	*NID2*	*SMAD9*	*ZEB2*
CTNNBIP1	*PTPRZ1*	*COPZ2*	*IL1R1*	*NOG*	*SNAI1*	
CTSZ	*RAB31*	*CRISPLD2*	*IL6R*	*NOTCH2*	*SPARC*	
DAB2	*RAPGEF5*	*CTNNB1*	*ITGA5*	*NUAK1*	*SPINT1*	
DSC2	*RUNX1*	*CXCR4*	*ITGB1*	*NUDT13*	*SRC*	
ELF5	*SERPINF1*	*DAPK1*	*KIF3C*	*OVOL2*	*ST6GALNAC2*	
EML1	*ST14*	*EGFR*	*KRT14*	*PAG1*	*STAT3*	
EZH2	*STAT5B*	*EMP3*	*KRT15*	*PDE4A*	*TAGLN*	
FAM169A	*TBX3*	*ENG*	*KRT19*	*PHLDA1*	*TBX2*	
FLT4	*TMEM30B*	*EPAS1*	*KRT7*	*PKP2*	*TBX20*	
FOXC1	*TPD52L1*	*ERBB2*	*LAD1*	*PMP22*	*TEAD1*	
FOXM1	*TWIST1*	*ETV4*	*LEP*	*PRKCE*	*TGFB1*	
FSCN1	*VSNL1*	*F11R*	*LGALS1*	*PRRG4*	*TGFB2*	
GMNN	*YAP1*	*FBLN5*	*LIMA1*	*PRSS8*	*TGM2*	
HBEGF		*FBN1*	*LIMS1*	*PTK*	*TIMP3*	
ID2		*FGFBP1*	*LIMS2*	*PTPN14*	*TNF*	
IL18		*FGFR1*	*LOXL1*	*RAC1*	*TPM1*	
JAG1		*FHL2*	*LOXL2*	*RCN3*	*TPM2*	
KRAS		*FLT1*	*LRRC15*	*RECK*	*VWCE*	
LAMA1		*FN1*	*LTBP2*	*RHOA*	*WWTR1*	
MAPK14		*FSTL1*	*MAPK3*	*RHOD*	*YBX1*	
MARVELD3		*FXYD3*	*MAPK8*	*RUSC2*		
