# Supplementary material for: Epigenetic Changes Regulating Epithelial–Mesenchymal Plasticity in Human Trophoblast Differentiation
Source: Cells. 2025 Jun 24;14(13):970. doi: 10.3390/cells14130970 (PMC12249213; doi:10.3390/cells14130970)
Supplement: Supplementary file 1 [file cells-14-00970-s001.zip › cells-3668026-supplementary/Table_S8.pdf]

Supplementary Table S8: Potential effects on EMP of altered expression of DE-EMT genes that show a gain of methylation

Gene ID	Fold change	Effect on EMT	References	Gene ID	Fold change	Effect on EMT	References
HSPG2	38.4	Pro-EMT	[1, 2]	*PTPN14*	-2.4	Pro-EMT	[3, 4]
ASCL2	37.3	Pro-EMT	[5-7]	*NOTCH1*	-3.0	Pro-MET	[8, 9]
LAIR2	31.7	Pro-EMT/Pro-MET	[10-13]	*TPD52L1*	-2.9	Pro-MET	[14, 15]
PMEPA1	27.0	Pro-EMT	[16, 17]	*MAP3K4*	-3.0	Pro-EMT	[18, 19]
TGFB1	17.5	Pro-EMT	[20, 21]	*TGM2*	-3.1	Pro-MET	[22, 23]
FLT4	13.9	Pro-EMT	[24, 25]	*SYK*	-3.7	Pro-MET/Pro-EMT	[26, 27]
MICALL2	12.5	Pro-EMT	[28, 29]	*IGF1R*	-4.2	Pro-MET	[30, 31]
LPCAT1	10.2	Pro-EMT	[32, 33]	*FOXK1*	-4.8	Pro-MET	[34, 35]
TWIST2	10.2	Pro-EMT/Pro-MET	[36-38]	*BCL2*	-6.9	Pro-MET	[39, 40]
COL5A1	9.9	Pro-EMT	[41, 42]	*MET*	-7.73	Pro-MET	[43, 44]
RUNX1	7.5	Pro-EMT/Pro-MET	[45-47]	*FBN2*	-12.3	Pro-MET	[48, 49]
FSCN1	6.2	Pro-EMT	[50, 51]	*FXYD3*	-13.1	Pro-MET/Pro-EMT	[52, 53]
COL4A2	4.4	Pro-EMT	[54, 55]	*SPINT1*	-14.3	Pro-MET/Pro-EMT	[56-58]
BMP1	3.8	Pro-EMT/Pro-MET	[59-61]	*ALDH1A3*	-14.8	Pro-MET	[62, 63]
PLOD1	3.5	Pro-EMT	[64, 65]	*JAG1*	-15.8	Pro-MET	[66, 67]
				FGFR2	-19.1	Pro-MET/Pro-EMT	[68-70]
				BMP7	-24.0	Pro-MET/Pro-EMT	[71-73]
				MSX2	-46.7	Pro-MET	[74, 75]
				LRP5	-91.4	Pro-MET	[76, 77]
				SVEP1	-93.7	Pro-EMT/Pro-MET	[28, 78, 79]
				ZBTB7C	-104.2	Pro-EMT/Pro-MET	[80-82]
				LARGE2	-114.9	Pro-EMT	[83, 84]
				NRP2	-123.5	Pro-MET	[85, 86]
				FRAS1	-128.1	Pro-EMT/Pro-MET	[87, 88]
				EPHB4	-140.9	Pro-EMT/Pro-MET	[89, 90]
				ACSS1	-189.9	Pro-MET	[91, 92]
				PEG10	-195.8	Pro-MET	[93, 94]
				SLC27A2	-250.0	Pro-MET/Pro-EMT	[95, 96]
				LRP2	-293.8	Pro-EMT/Pro-MET	[97, 98]

Pro-EMT: Change which has the effect of promoting the EMT/suppressing MET
Pro-MET: Change which has the effect of suppressing the EMT/promoting MET
Pro-EMT/Pro-MET: May have the effect of promoting EMT or MET, depending on context

NB: The effects shown above are the effect of the change in expression of that gene on the shift between epithelial and mesenchymal phenotypes. Thus reduction in expression of a gene which promotes EMT for example is regarded as Pro-MET

References

1. Kalscheuer S, Khanna V, Kim H, Li S, Sachdev D, DeCarlo A, et al. Discovery of HSPG2 (Perlecan) as a Therapeutic Target in Triple Negative Breast Cancer. Sci Rep. 2019;9(1):12492. Epub 20190828. doi: 10.1038/s41598-019-48993-6. PubMed PMID: 31462656; PubMed Central PMCID:PMC6713791.

2. Warren CR, Grindel BJ, Francis L, Carson DD, Farach-Carson MC. Transcriptional activation by NFkappaB increases perlecan/HSPG2 expression in the desmoplastic prostate tumor microenvironment. Journal of cellular biochemistry. 2014;115(7):1322-33. doi: 10.1002/jcb.24788. PubMed PMID: 24700612; PubMed Central PMCID:PMC4091977.

3. Liu X, Yang N, Figel SA, Wilson KE, Morrison CD, Gelman IH, et al. PTPN14 interacts with and negatively regulates the oncogenic function of YAP. Oncogene. 2013;32(10):1266-73. Epub 20120423. doi: 10.1038/onc.2012.147. PubMed PMID: 22525271; PubMed Central PMCID:PMC4402938.

4. Belle L, Ali N, Lonic A, Li X, Paltridge JL, Roslan S, et al. The tyrosine phosphatase PTPN14 (Pez) inhibits metastasis by altering protein trafficking. Sci Signal. 2015;8(364):ra18. Epub 20150217. doi: 10.1126/scisignal.2005547. PubMed PMID: 25690013.

5. Tian Y, Pan Q, Shang Y, Zhu R, Ye J, Liu Y, et al. MicroRNA-200 (miR-200) cluster regulation by achaete scute-like 2 (Ascl2): impact on the epithelial-mesenchymal transition in colon cancer cells. J Biol Chem. 2014;289(52):36101-15. Epub 20141104. doi: 10.1074/jbc.M114.598383. PubMed PMID: 25371200; PubMed Central PMCID:PMC4276874.

6. Varberg KM, Iqbal K, Muto M, Simon ME, Scott RL, Kozai K, et al. ASCL2 reciprocally controls key trophoblast lineage decisions during hemochorial placenta development. Proc Natl Acad Sci U S A. 2021;118(10). doi: 10.1073/pnas.2016517118. PubMed PMID: 33649217; PubMed Central PMCID:PMC7958375.

7. Zuo Q, Wang J, Chen C, Zhang Y, Feng DX, Zhao R, et al. ASCL2 expression contributes to gastric tumor migration and invasion by downregulating miR223 and inducing EMT. Mol Med Rep. 2018;18(4):3751-9. Epub 20180808. doi: 10.3892/mmr.2018.9363. PubMed PMID: 30106147; PubMed Central PMCID:PMC6131580.

8. Natsuizaka M, Whelan KA, Kagawa S, Tanaka K, Giroux V, Chandramouleeswaran PM, et al. Interplay between Notch1 and Notch3 promotes EMT and tumor initiation in squamous cell carcinoma. Nat Commun. 2017;8(1):1758. Epub 20171124. doi: 10.1038/s41467-017-01500-9. PubMed PMID: 29170450; PubMed Central PMCID:PMC5700926.

9. Shao S, Zhao X, Zhang X, Luo M, Zuo X, Huang S, et al. Notch1 signaling regulates the epithelial-mesenchymal transition and invasion of breast cancer in a Slug-dependent manner. Mol Cancer. 2015;14(1):28. Epub 20150203. doi: 10.1186/s12943-015-0295-3. PubMed PMID: 25645291; PubMed Central PMCID:PMC4322803.

10. Wang P, Zhuang W, Zheng Z, Zhang L, Zhang X, Chen Q. Dissecting T-cell heterogeneity in esophageal squamous cell carcinoma reveals the potential role of LAIR2 in antitumor immunity. Clin Exp Immunol. 2023;214(1):36-49. doi: 10.1093/cei/uxad073. PubMed PMID: 37422711; PubMed Central PMCID:PMC10711353.

11. Ly D, Li Q, Navab R, Zeltz C, Fang L, Cabanero M, et al. Tumor-Associated Regulatory T Cell Expression of LAIR2 Is Prognostic in Lung Adenocarcinoma. Cancers (Basel). 2021;14(1). Epub 20211231. doi: 10.3390/cancers14010205. PubMed PMID: 35008369; PubMed Central PMCID:PMC8744930.

12. Farah O, Nguyen C, Tekkatte C, Parast MM. Trophoblast lineage-specific differentiation and associated alterations in preeclampsia and fetal growth restriction. Placenta. 2020;102:4-9. Epub 20200213. doi: 10.1016/j.placenta.2020.02.007. PubMed PMID: 33218578; PubMed Central PMCID:PMC7680505.

13. Choi JH, Lee BS, Jang JY, Lee YS, Kim HJ, Roh J, et al. Single-cell transcriptome profiling of the stepwise progression of head and neck cancer. Nat Commun. 2023;14(1):1055. Epub 20230224. doi: 10.1038/s41467-023-36691-x. PubMed PMID: 36828832; PubMed Central PMCID:PMC9958029.

14. Boutros R, Byrne JA. D53 (TPD52L1) is a cell cycle-regulated protein maximally expressed at the G2-M transition in breast cancer cells. Experimental cell research. 2005;310(1):152-65. doi: 10.1016/j.yexcr.2005.07.009. PubMed PMID: 16112108.

15. Hong Q, Li B, Cai X, Lv Z, Cai S, Zhong Y, et al. Transcriptomic Analyses of the Adenoma-Carcinoma Sequence Identify Hallmarks Associated With the Onset of Colorectal Cancer. Front Oncol. 2021;11:704531. Epub 20210811. doi: 10.3389/fonc.2021.704531. PubMed PMID: 34458146; PubMed Central PMCID:PMC8387103.

16. Zhang L, Wang X, Lai C, Zhang H, Lai M. PMEPA1 induces EMT via a non-canonical TGF-beta signalling in colorectal cancer. J Cell Mol Med. 2019;23(5):3603-15. Epub 20190319. doi: 10.1111/jcmm.14261. PubMed PMID: 30887697; PubMed Central PMCID:PMC6484414.

17. Zhu Q, Wang Y, Liu Y, Yang X, Shuai Z. Prostate transmembrane androgen inducible protein 1 (PMEPA1): regulation and clinical implications. Front Oncol. 2023;13:1298660. Epub 20231220. doi: 10.3389/fonc.2023.1298660. PubMed PMID: 38173834; PubMed Central PMCID:PMC10761476.

18. Mobley RJ, Raghu D, Duke LD, Abell-Hart K, Zawistowski JS, Lutz K, et al. MAP3K4 Controls the Chromatin Modifier HDAC6 during Trophoblast Stem Cell Epithelial-to-Mesenchymal Transition. Cell reports. 2017;18(10):2387-400. doi: 10.1016/j.celrep.2017.02.030. PubMed PMID: 28273454; PubMed Central PMCID:PMC5496714.

19. Shendy NAM, Raghu D, Roy S, Perry CH, Safi A, Branco MR, et al. Coordinated regulation of Rel expression by MAP3K4, CBP, and HDAC6 controls phenotypic switching. Commun Biol. 2020;3(1):475. Epub 20200828. doi: 10.1038/s42003-020-01200-z. PubMed PMID: 32859943; PubMed Central PMCID:PMC7455715.

20. Hao Y, Baker D, Ten Dijke P. TGF-beta-Mediated Epithelial-Mesenchymal Transition and Cancer Metastasis. Int J Mol Sci. 2019;20(11). Epub 20190605. doi: 10.3390/ijms20112767. PubMed PMID: 31195692; PubMed Central PMCID:PMC6600375.

21. Pang MF, Georgoudaki AM, Lambut L, Johansson J, Tabor V, Hagikura K, et al. TGF-beta1-induced EMT promotes targeted migration of breast cancer cells through the lymphatic system by the activation of CCR7/CCL21-mediated chemotaxis. Oncogene. 2016;35(6):748-60. Epub 20150511. doi: 10.1038/onc.2015.133. PubMed PMID: 25961925; PubMed Central PMCID:PMC4753256.

22. He W, Sun Z, Liu Z. Silencing of TGM2 reverses epithelial to mesenchymal transition and modulates the chemosensitivity of breast cancer to docetaxel. Exp Ther Med. 2015;10(4):1413-8. Epub 20150812. doi: 10.3892/etm.2015.2679. PubMed PMID: 26622499; PubMed Central PMCID:PMC4577978.

23. Ma H, Xie L, Zhang L, Yin X, Jiang H, Xie X, et al. Activated hepatic stellate cells promote epithelial-to-mesenchymal transition in hepatocellular carcinoma through transglutaminase 2-induced pseudohypoxia. Commun Biol. 2018;1:168. Epub 20181025. doi: 10.1038/s42003-018-0177-5. PubMed PMID: 30393774; PubMed Central PMCID:PMC6202353.

24. Kurmyshkina O, Kovchur P, Schegoleva L, Volkova T. Markers of Angiogenesis, Lymphangiogenesis, and Epithelial-Mesenchymal Transition (Plasticity) in CIN and Early Invasive Carcinoma of the Cervix: Exploring Putative Molecular Mechanisms Involved in Early Tumor Invasion. Int J Mol Sci. 2020;21(18). Epub 20200906. doi: 10.3390/ijms21186515. PubMed PMID: 32899940; PubMed Central PMCID:PMC7554870.

25. Kong D, Zhou H, Neelakantan D, Hughes CJ, Hsu JY, Srinivasan RR, et al. VEGF-C mediates tumor growth and metastasis through promoting EMT-epithelial breast cancer cell crosstalk. Oncogene. 2021;40(5):964-79. Epub 20201209. doi: 10.1038/s41388-020-01539-x. PubMed PMID: 33299122; PubMed Central PMCID:PMC7867573.

26. Geahlen RL. Getting Syk: spleen tyrosine kinase as a therapeutic target. Trends Pharmacol Sci. 2014;35(8):414-22. Epub 20140626. doi: 10.1016/j.tips.2014.05.007. PubMed PMID: 24975478; PubMed Central PMCID:PMC4119858.

27. Krisenko MO, Geahlen RL. Calling in SYK: SYK's dual role as a tumor promoter and tumor suppressor in cancer. Biochim Biophys Acta. 2015;1853(1):254-63. Epub 20141104. doi: 10.1016/j.bbamcr.2014.10.022. PubMed PMID: 25447675; PubMed Central PMCID:PMC4254495.

28. Chen L, He Y, Han Z, Gong W, Tian X, Guo L, et al. The impact of decreased expression of SVEP1 on abnormal neovascularization and poor prognosis in patients with intrahepatic cholangiocarcinoma. Front Genet. 2022;13:1127753. Epub 20230109. doi: 10.3389/fgene.2022.1127753. PubMed PMID: 36699464; PubMed Central PMCID:PMC9870246.

29. Ding J, Huang F, Wu G, Han T, Xu F, Weng D, et al. MiR-519d-3p suppresses invasion and migration of trophoblast cells via targeting MMP-2. PLoS ONE [Electronic Resource]. 2015;10(3):e0120321. PubMed PMID: 25803859.

30. Alfaro-Arnedo E, Lopez IP, Pineiro-Hermida S, Canalejo M, Gotera C, Sola JJ, et al. IGF1R acts as a cancer-promoting factor in the tumor microenvironment facilitating lung metastasis implantation and progression. Oncogene. 2022;41(28):3625-39. Epub 20220610. doi: 10.1038/s41388-022-02376-w. PubMed PMID: 35688943; PubMed Central PMCID:PMC9184253.

31. Vazquez-Martin A, Cufi S, Oliveras-Ferraros C, Torres-Garcia VZ, Corominas-Faja B, Cuyas E, et al. IGF-1R/epithelial-to-mesenchymal transition (EMT) crosstalk suppresses the erlotinib-sensitizing effect of EGFR exon 19 deletion mutations. Sci Rep. 2013;3:2560. doi: 10.1038/srep02560. PubMed PMID: 23994953; PubMed Central PMCID:PMC3759044.

32. Shen L, Gu P, Qiu C, Ding WT, Zhang L, Cao WY, et al. Lysophosphatidylcholine acyltransferase 1 promotes epithelial-mesenchymal transition of hepatocellular carcinoma via the Wnt/beta-catenin signaling pathway. Ann Hepatol. 2022;27(3):100680. Epub 20220130. doi: 10.1016/j.aohep.2022.100680. PubMed PMID: 35108614.

33. Bi J, Ichu TA, Zanca C, Yang H, Zhang W, Gu Y, et al. Oncogene Amplification in Growth Factor Signaling Pathways Renders Cancers Dependent on Membrane Lipid Remodeling. Cell Metab. 2019;30(3):525-38 e8. Epub 20190711. doi: 10.1016/j.cmet.2019.06.014. PubMed PMID: 31303424; PubMed Central PMCID:PMC6742496.

34. Liu Y, Ding W, Ge H, Ponnusamy M, Wang Q, Hao X, et al. FOXK transcription factors: Regulation and critical role in cancer. Cancer Lett. 2019;458:1-12. Epub 20190524. doi: 10.1016/j.canlet.2019.05.030. PubMed PMID: 31132431.

35. Zhang H, Wu X, Xiao Y, Wu L, Peng Y, Tang W, et al. Coexpression of FOXK1 and vimentin promotes EMT, migration, and invasion in gastric cancer cells. J Mol Med (Berl). 2019;97(2):163-76. Epub 20181127. doi: 10.1007/s00109-018-1720-z. PubMed PMID: 30483822.

36. Ansieau S, Bastid J, Doreau A, Morel AP, Bouchet BP, Thomas C, et al. Induction of EMT by twist proteins as a collateral effect of tumor-promoting inactivation of premature senescence. Cancer Cell. 2008;14(1):79-89. doi: 10.1016/j.ccr.2008.06.005. PubMed PMID: 18598946.

37. Fang X, Cai Y, Liu J, Wang Z, Wu Q, Zhang Z, et al. Twist2 contributes to breast cancer progression by promoting an epithelial-mesenchymal transition and cancer stem-like cell self-renewal. Oncogene. 2011;30(47):4707-20. Epub 20110523. doi: 10.1038/onc.2011.181. PubMed PMID: 21602879.

38. Ishikawa T, Shimizu T, Ueki A, Yamaguchi SI, Onishi N, Sugihara E, et al. Twist2 functions as a tumor suppressor in murine osteosarcoma cells. Cancer Sci. 2013;104(7):880-8. Epub 20130509. doi: 10.1111/cas.12163. PubMed PMID: 23557174; PubMed Central PMCID:PMC7657229.

39. Zuo J, Ishikawa T, Boutros S, Xiao Z, Humtsoe JO, Kramer RH. Bcl-2 overexpression induces a partial epithelial to mesenchymal transition and promotes squamous carcinoma cell invasion and metastasis. Mol Cancer Res. 2010;8(2):170-82. Epub 20100209. doi: 10.1158/1541-7786.MCR-09-0354. PubMed PMID: 20145039.

40. Du C, Zhang X, Yao M, Lv K, Wang J, Chen L, et al. Bcl-2 promotes metastasis through the epithelial-to-mesenchymal transition in the BCap37 medullary breast cancer cell line. Oncol Lett. 2018;15(6):8991-898. Epub 20180410. doi: 10.3892/ol.2018.8455. PubMed PMID: 29844816; PubMed Central PMCID:PMC5958888.

41. Liu W, Wei H, Gao Z, Chen G, Liu Y, Gao X, et al. COL5A1 may contribute the metastasis of lung adenocarcinoma. Gene. 2018;665:57-66. Epub 20180424. doi: 10.1016/j.gene.2018.04.066. PubMed PMID: 29702185.

42. Tsai HF, Chang YC, Li CH, Chan MH, Chen CL, Tsai WC, et al. Type V collagen alpha 1 chain promotes the malignancy of glioblastoma through PPRC1-ESM1 axis activation and extracellular matrix remodeling. Cell Death Discov. 2021;7(1):313. Epub 20211026. doi: 10.1038/s41420-021-00661-3. PubMed PMID: 34702798; PubMed Central PMCID:PMC8548600.

43. Wang S, Ma H, Yan Y, Chen Y, Fu S, Wang J, et al. cMET promotes metastasis and epithelial-mesenchymal transition in colorectal carcinoma by repressing RKIP. Journal of cellular physiology. 2021;236(5):3963-78. Epub 20201105. doi: 10.1002/jcp.30142. PubMed PMID: 33151569.

44. Zhang Y, Xia M, Jin K, Wang S, Wei H, Fan C, et al. Function of the c-Met receptor tyrosine kinase in carcinogenesis and associated therapeutic opportunities. Mol Cancer. 2018;17(1):45. Epub 20180219. doi: 10.1186/s12943-018-0796-y. PubMed PMID: 29455668; PubMed Central PMCID:PMC5817860.

45. Khawaled S, Aqeilan RI. RUNX1, a new regulator of EMT in breast cancer. Oncotarget. 2017;8(11):17407-8. doi: 10.18632/oncotarget.15623. PubMed PMID: 28407696; PubMed Central PMCID:PMC5392256.

46. Zhou T, Luo M, Cai W, Zhou S, Feng D, Xu C, et al. Runt-Related Transcription Factor 1 (RUNX1) Promotes TGF-beta-Induced Renal Tubular Epithelial-to-Mesenchymal Transition (EMT) and Renal Fibrosis through the PI3K Subunit p110delta. EBioMedicine. 2018;31:217-25. Epub 20180511. doi: 10.1016/j.ebiom.2018.04.023. PubMed PMID: 29759484; PubMed Central PMCID:PMC6013935.

47. Li Q, Lai Q, He C, Fang Y, Yan Q, Zhang Y, et al. RUNX1 promotes tumour metastasis by activating the Wnt/beta-catenin signalling pathway and EMT in colorectal cancer. J Exp Clin Cancer Res. 2019;38(1):334. Epub 20190801. doi: 10.1186/s13046-019-1330-9. PubMed PMID: 31370857; PubMed Central PMCID:PMC6670220.

48. Wang J, Akter R, Shahriar MF, Uddin MN. Cancer-Associated Stromal Fibroblast-Derived Transcriptomes Predict Poor Clinical Outcomes and Immunosuppression in Colon Cancer. Pathol

Oncol Res. 2022;28:1610350. Epub 20220804. doi: 10.3389/pore.2022.1610350. PubMed PMID: 35991839; PubMed Central PMCID:PMC9385976.

49. Stavely R, Hotta R, Guyer RA, Picard N, Rahman AA, Omer M, et al. A distinct transcriptome characterizes neural crest-derived cells at the migratory wavefront during enteric nervous system development. Development. 2023;150(5). Epub 20230306. doi: 10.1242/dev.201090. PubMed PMID: 36779913; PubMed Central PMCID:PMC10108706.

50. Li J, Zhang S, Pei M, Wu L, Liu Y, Li H, et al. FSCN1 Promotes Epithelial-Mesenchymal Transition Through Increasing Snail1 in Ovarian Cancer Cells. Cell Physiol Biochem. 2018;49(5):1766-77. Epub 20180919. doi: 10.1159/000493622. PubMed PMID: 30231243.

51. Li Z, Shi J, Zhang N, Zheng X, Jin Y, Wen S, et al. FSCN1 acts as a promising therapeutic target in the blockade of tumor cell motility: a review of its function, mechanism, and clinical significance. J Cancer. 2022;13(8):2528-39. Epub 20220509. doi: 10.7150/jca.67977. PubMed PMID: 35711849; PubMed Central PMCID:PMC9174856.

52. Yamamoto H, Mukaisho K, Sugihara H, Hattori T, Asano S. Down-regulation of FXYD3 is induced by transforming growth factor-beta signaling via ZEB1/deltaEF1 in human mammary epithelial cells. Biol Pharm Bull. 2011;34(3):324-9. doi: 10.1248/bpb.34.324. PubMed PMID: 21372379.

53. Kayed H, Kleeff J, Kolb A, Ketterer K, Keleg S, Felix K, et al. FXYD3 is overexpressed in pancreatic ductal adenocarcinoma and influences pancreatic cancer cell growth. Int J Cancer. 2006;118(1):43-54. doi: 10.1002/ijc.21257. PubMed PMID: 16003754.

54. Guo Y, Yuan Z, Hu Z, Gao Y, Guo H, Zhu H, et al. Diagnostic model constructed by five EMT-related genes for renal fibrosis and reflecting the condition of immune-related cells. Frontiers in immunology. 2023;14:1161436. Epub 20230517. doi: 10.3389/fimmu.2023.1161436. PubMed PMID: 37266443; PubMed Central PMCID:PMC10229861.

55. Quenneville J, Feghaly A, Tual M, Thomas K, Major F, Gagnon E. Long-term severe hypoxia adaptation induces non-canonical EMT and a novel Wilms Tumor 1 (WT1) isoform. Cancer Gene Ther. 2024;31(8):1237-50. Epub 20240708. doi: 10.1038/s41417-024-00795-3. PubMed PMID: 38977895; PubMed Central PMCID:PMC11327107.

56. Cheng H, Fukushima T, Takahashi N, Tanaka H, Kataoka H. Hepatocyte growth factor activator inhibitor type 1 regulates epithelial to mesenchymal transition through membrane-bound serine proteinases. Cancer research. 2009;69(5):1828-35. Epub 20090217. doi: 10.1158/0008-5472.CAN-08-3728. PubMed PMID: 19223533.

57. Gomez-Abenza E, Ibanez-Molero S, Garcia-Moreno D, Fuentes I, Zon LI, Mione MC, et al. Zebrafish modeling reveals that SPINT1 regulates the aggressiveness of skin cutaneous melanoma and its crosstalk with tumor immune microenvironment. J Exp Clin Cancer Res. 2019;38(1):405. Epub 20190913. doi: 10.1186/s13046-019-1389-3. PubMed PMID: 31519199; PubMed Central PMCID:PMC6743187.

58. Liu CL, Yang PS, Chien MN, Chang YC, Lin CH, Cheng SP. Expression of serine peptidase inhibitor Kunitz type 1 in differentiated thyroid cancer. Histochem Cell Biol. 2018;149(6):635-44. Epub 20180312. doi: 10.1007/s00418-018-1660-2. PubMed PMID: 29532159.

59. Xiao W, Wang X, Wang T, Xing J. Overexpression of BMP1 reflects poor prognosis in clear cell renal cell carcinoma. Cancer Gene Ther. 2020;27(5):330-40. Epub 20190603. doi: 10.1038/s41417-019-0107-9. PubMed PMID: 31155610; PubMed Central PMCID:PMC7237353.

60. Zhu X, Luo X, Jiang S, Wang H. Bone Morphogenetic Protein 1 Targeting COL1A1 and COL1A2 to Regulate the Epithelial-Mesenchymal Transition Process of Colon Cancer SW620 Cells. J Nanosci Nanotechnol. 2020;20(3):1366-74. doi: 10.1166/jnn.2020.17362. PubMed PMID: 31492296.

61. Prieto TG, Baldavira CM, Machado-Rugolo J, Farhat C, Olivieri EHR, de Sa VK, et al. Pulmonary Neuroendocrine Neoplasms Overexpressing Epithelial-Mesenchymal Transition Mechanical Barriers Genes Lack Immune-Suppressive Response and Present an Increased Risk of Metastasis. Front Oncol. 2021;11:645623. Epub 20210830. doi: 10.3389/fonc.2021.645623. PubMed PMID: 34527572; PubMed Central PMCID:PMC8435885.

62. McLean ME, MacLean MR, Cahill HF, Arun RP, Walker OL, Wasson MD, et al. The Expanding Role of Cancer Stem Cell Marker ALDH1A3 in Cancer and Beyond. Cancers (Basel). 2023;15(2). Epub 20230113. doi: 10.3390/cancers15020492. PubMed PMID: 36672441; PubMed Central PMCID:PMC9857290.

63. Yamashita D, Minata M, Ibrahim AN, Yamaguchi S, Coviello V, Bernstock JD, et al. Identification of ALDH1A3 as a viable therapeutic target in breast cancer metastasis-initiating cells. Molecular Cancer Therapeutics. 2020:molcanther.0461.2019. doi: 10.1158/1535-7163.MCT-19-0461.

64. Qi Y, Xu R. Roles of PLODs in Collagen Synthesis and Cancer Progression. Front Cell Dev Biol. 2018;6:66. Epub 20180628. doi: 10.3389/fcell.2018.00066. PubMed PMID: 30003082; PubMed Central PMCID:PMC6031748.

65. Wang Z, Shi Y, Ying C, Jiang Y, Hu J. Hypoxia-induced PLOD1 overexpression contributes to the malignant phenotype of glioblastoma via NF-kappaB signaling. Oncogene. 2021;40(8):1458-75. Epub 20210108. doi: 10.1038/s41388-020-01635-y. PubMed PMID: 33420370; PubMed Central PMCID:PMC7906902.

66. Negri F, Bottarelli L, Pedrazzi G, Maddalo M, Leo L, Milanese G, et al. Notch-Jagged1 signaling and response to bevacizumab therapy in advanced colorectal cancer: A glance to radiomics or back to physiopathology? Front Oncol. 2023;13:1132564. Epub 20230228. doi: 10.3389/fonc.2023.1132564. PubMed PMID: 36925919; PubMed Central PMCID:PMC10011088.

67. Xiu MX, Liu YM, Kuang BH. The oncogenic role of Jagged1/Notch signaling in cancer. Biomed Pharmacother. 2020;129:110416. Epub 20200625. doi: 10.1016/j.biopha.2020.110416. PubMed PMID: 32593969.

68. Xu Y, Gao F, Zhang J, Cai P, Xu D. Fibroblast growth factor receptor 2 promotes the proliferation, migration, and invasion of ectopic stromal cells via activation of extracellular-signal-regulated kinase signaling pathway in endometriosis. Bioengineered. 2022;13(4):8360-71. doi: 10.1080/21655979.2022.2054207. PubMed PMID: 35311468; PubMed Central PMCID:PMC9161834.

69. Lei JH, Lee MH, Miao K, Huang Z, Yao Z, Zhang A, et al. Activation of FGFR2 Signaling Suppresses BRCA1 and Drives Triple-Negative Mammary Tumorigenesis That is Sensitive to Immunotherapy. Adv Sci (Weinh). 2021;8(21):e2100974. Epub 20210913. doi: 10.1002/advs.202100974. PubMed PMID: 34514747; PubMed Central PMCID:PMC8564435.

70. Grygielewicz P, Dymek B, Bujak A, Gunerka P, Stanczak A, Lamparska-Przybysz M, et al. Epithelial-mesenchymal transition confers resistance to selective FGFR inhibitors in SNU-16 gastric cancer cells. Gastric Cancer. 2016;19(1):53-62. Epub 20141119. doi: 10.1007/s10120-014-0444-1. PubMed PMID: 25407459; PubMed Central PMCID:PMC4688307.

71. Sun R, Guan H, Liu W, Liang J, Wang F, Li C. Expression of BMP7 in cervical cancer and inhibition of epithelial-mesenchymal transition by BMP7 knockdown in HeLa cells. Int J Mol Med. 2020;45(5):1417-24. Epub 20200227. doi: 10.3892/ijmm.2020.4519. PubMed PMID: 32323730; PubMed Central PMCID:PMC7138274.

72. Ying X, Sun Y, He P. Bone Morphogenetic Protein-7 Inhibits EMT-Associated Genes in Breast Cancer. Cell Physiol Biochem. 2015;37(4):1271-8. Epub 20151005. doi: 10.1159/000430249. PubMed PMID: 26431436.

73. Zeisberg M, Hanai J, Sugimoto H, Mammoto T, Charytan D, Strutz F, et al. BMP-7 counteracts TGF-beta1-induced epithelial-to-mesenchymal transition and reverses chronic renal injury. Nature medicine. 2003;9(7):964-8. doi: 10.1038/nm888. PubMed PMID: 12808448.

74. Liang H, Zhang Q, Lu J, Yang G, Tian N, Wang X, et al. MSX2 Induces Trophoblast Invasion in Human Placenta. PLoS ONE [Electronic Resource]. 2016;11(4):e0153656. PubMed PMID: 27088357.

75. Zhai Y, Iura A, Yeasmin S, Wiese AB, Wu R, Feng Y, et al. MSX2 is an oncogenic downstream target of activated WNT signaling in ovarian endometrioid adenocarcinoma. Oncogene. 2011;30(40):4152-62. Epub 20110418. doi: 10.1038/onc.2011.123. PubMed PMID: 21499300; PubMed Central PMCID:PMC3140605.

76. Hong J, Xie Z, Yang Z, Yang F, Liao H, Rao S, et al. Inactivation of Wnt-LRP5 signaling suppresses the proliferation and migration of ovarian cancer cells. Transl Cancer Res. 2021;10(5):2277-85. doi: 10.21037/tcr-20-3462. PubMed PMID: 35116545; PubMed Central PMCID:PMC8797788.

77. Xue W, Yang L, Chen C, Ashrafizadeh M, Tian Y, Sun R. Wnt/beta-catenin-driven EMT regulation in human cancers. Cell Mol Life Sci. 2024;81(1):79. Epub 20240209. doi: 10.1007/s00018-023-05099-7. PubMed PMID: 38334836; PubMed Central PMCID:PMC10857981.

78. Chen L, Liu D, Yi X, Qi L, Tian X, Sun B, et al. The novel miR-1269b-regulated protein SVEP1 induces hepatocellular carcinoma proliferation and metastasis likely through the PI3K/Akt pathway. Cell Death Dis. 2020;11(5):320. Epub 20200505. doi: 10.1038/s41419-020-2535-8. PubMed PMID: 32371982; PubMed Central PMCID:PMC7200779.

79. Yang J, Deng Q, Chen Z, Chen Y, Fu Z. BVES-AS1 suppresses the colorectal cancer progression via the miR-1269a/b-SVEP1-PI3K/AKT axis. Adv Clin Exp Med. 2024;33(11):1217-36. doi: 10.17219/acem/175050. PubMed PMID: 38239081.

80. Guo C, Xiong D, Yang B, Zhang H, Gu W, Liu M, et al. The expression and clinical significance of ZBTB7 in transitional cell carcinoma of the bladder. Oncol Lett. 2017;14(4):4857-62. Epub 20170824. doi: 10.3892/ol.2017.6814. PubMed PMID: 29085492; PubMed Central PMCID:PMC5649710.

81. Chen X, Jiang Z, Pu Y, Jiang X, Xiang L, Jiang Z. Zinc finger and BTB domain-containing 7C (ZBTB7C) expression as an independent prognostic factor for colorectal cancer and its relevant molecular mechanisms. Am J Transl Res. 2020;12(8):4141-59. Epub 20200815. PubMed PMID: 32913494; PubMed Central PMCID:PMC7476137.

82. Chen X, Jiang Z, Wang Z, Jiang Z. The prognostic and immunological effects of ZBTB7C across cancers: friend or foe? Aging (Albany NY). 2021;13(9):12849-64. Epub 20210504. doi: 10.18632/aging.202955. PubMed PMID: 33946045; PubMed Central PMCID:PMC8148469.

83. Miller MR, Ma D, Schappet J, Breheny P, Mott SL, Bannick N, et al. Downregulation of dystroglycan glycosyltransferases LARGE2 and ISPD associate with increased mortality in clear cell renal cell carcinoma. Mol Cancer. 2015;14:141. Epub 20150730. doi: 10.1186/s12943-015-0416-z. PubMed PMID: 26220087; PubMed Central PMCID:PMC4518861.

84. Huang Q, Miller MR, Schappet J, Henry MD. The glycosyltransferase LARGE2 is repressed by Snail and ZEB1 in prostate cancer. Cancer Biol Ther. 2015;16(1):125-36. doi: 10.4161/15384047.2014.987078. PubMed PMID: 25455932; PubMed Central PMCID:PMC4623020.

85. Islam R, Mishra J, Bodas S, Bhattacharya S, Batra SK, Dutta S, et al. Role of Neuropilin-2-mediated signaling axis in cancer progression and therapy resistance. Cancer Metastasis Rev. 2022;41(3):771-87. Epub 20220701. doi: 10.1007/s10555-022-10048-0. PubMed PMID: 35776228; PubMed Central PMCID:PMC9247951.

86. Gemmill RM, Nasarre P, Nair-Menon J, Cappuzzo F, Landi L, D'Incecco A, et al. The neuropilin 2 isoform NRP2b uniquely supports TGFbeta-mediated progression in lung cancer. Sci Signal. 2017;10(462). Epub 20170117. doi: 10.1126/scisignal.aag0528. PubMed PMID: 28096505; PubMed Central PMCID:PMC5969810.

87. Wang G, Wang Z, Lu H, Zhao Z, Guo L, Kong F, et al. Comprehensive analysis of FRAS1/FREM family as potential biomarkers and therapeutic targets in renal clear cell carcinoma. Frontiers in pharmacology. 2022;13:972934. Epub 20220929. doi: 10.3389/fphar.2022.972934. PubMed PMID: 36249757; PubMed Central PMCID:PMC9558830.

88. Umeda S, Kanda M, Miwa T, Tanaka H, Tanaka C, Kobayashi D, et al. Fraser extracellular matrix complex subunit 1 promotes liver metastasis of gastric cancer. Int J Cancer. 2020;146(10):2865-76. Epub 20191023. doi: 10.1002/ijc.32705. PubMed PMID: 31597194.

89. Abdelazeem KNM, Nguyen D, Corbo S, Darragh LB, Matsumoto MW, Van Court B, et al. Manipulating the EphB4-ephrinB2 axis to reduce metastasis in HNSCC. Oncogene. 2025;44(3):130-46. Epub 20241103. doi: 10.1038/s41388-024-03208-9. PubMed PMID: 39489818; PubMed Central PMCID:PMC11725500.

90. Chen L, Zhu S, Liu T, Zhao X, Xiang T, Hu X, et al. Aberrant epithelial cell interaction promotes esophageal squamous-cell carcinoma development and progression. Signal Transduct Target Ther. 2023;8(1):453. Epub 20231215. doi: 10.1038/s41392-023-01710-2. PubMed PMID: 38097539; PubMed Central PMCID:PMC10721848.

91. Sun X, Zhang J, Nie Q. Inferring latent temporal progression and regulatory networks from cross-sectional transcriptomic data of cancer samples. PLoS Comput Biol. 2021;17(3):e1008379. Epub 20210305. doi: 10.1371/journal.pcbi.1008379. PubMed PMID: 33667222; PubMed Central PMCID:PMC7968745.

92. Hlavaty SI, Salcido KN, Pniewski KA, Mukha D, Ma W, Kannan T, et al. ACSS1-dependent acetate utilization rewires mitochondrial metabolism to support AML and melanoma tumor growth and metastasis. Cell reports. 2024;43(12):114988. Epub 20241121. doi: 10.1016/j.celrep.2024.114988. PubMed PMID: 39579354; PubMed Central PMCID:PMC11669533.

93. Zhang M, Sui C, Dai B, Shen W, Lu J, Yang J. PEG10 is imperative for TGF-beta1-induced epithelial-mesenchymal transition in hepatocellular carcinoma. Oncol Rep. 2017;37(1):510-8. Epub 20161129. doi: 10.3892/or.2016.5282. PubMed PMID: 28004118.

94. Xie T, Pan S, Zheng H, Luo Z, Tembo KM, Jamal M, et al. PEG10 as an oncogene: expression regulatory mechanisms and role in tumor progression. Cancer Cell Int. 2018;18:112. Epub 20180813. doi: 10.1186/s12935-018-0610-3. PubMed PMID: 30123090; PubMed Central PMCID:PMC6090666.

95. Xu N, Xiao W, Meng X, Li W, Wang X, Zhang X, et al. Up-regulation of SLC27A2 suppresses the proliferation and invasion of renal cancer by down-regulating CDK3-mediated EMT. Cell Death Discov. 2022;8(1):351. Epub 20220804. doi: 10.1038/s41420-022-01145-8. PubMed PMID: 35927229; PubMed Central PMCID:PMC9352701.

96. Veglia F, Tyurin VA, Blasi M, De Leo A, Kossenkov AV, Donthireddy L, et al. Fatty acid transport protein 2 reprograms neutrophils in cancer. Nature. 2019;569(7754):73-8. Epub 20190417. doi: 10.1038/s41586-019-1118-2. PubMed PMID: 30996346; PubMed Central PMCID:PMC6557120.

97. Andersen RK, Hammer K, Hager H, Christensen JN, Ludvigsen M, Honore B, et al. Melanoma tumors frequently acquire LRP2/megalin expression, which modulates melanoma cell proliferation and survival rates. Pigment Cell Melanoma Res. 2015;28(3):267-80. Epub 20150213. doi: 10.1111/pcmr.12352. PubMed PMID: 25585665.

98. Rasmussen MQ, Tindbaek G, Nielsen MM, Merrild C, Steiniche T, Pedersen JS, et al. Epigenetic Silencing of LRP2 Is Associated with Dedifferentiation and Poor Survival in Multiple Solid Tumor Types. Cancers (Basel). 2023;15(6). Epub 20230317. doi: 10.3390/cancers15061830. PubMed PMID: 36980716; PubMed Central PMCID:PMC10046670.
